# Gut microbiota-derived metabolites confer protection against SARS-CoV-2 infection

**DOI:** 10.1080/19490976.2022.2105609

**Published:** 2022-08-01

**Authors:** Julia A. Brown, Katherine Z. Sanidad, Serena Lucotti, Carolin M. Lieber, Robert M. Cox, Aparna Ananthanarayanan, Srijani Basu, Justin Chen, Mengrou Shan, Mohammed Amir, Fabian Schmidt, Yiska Weisblum, Michele Cioffi, Tingting Li, Florencia Madorsky Rowdo, M. Laura Martin, Chun-Jun Guo, Costas Lyssiotis, Brian T. Layden, Andrew J. Dannenberg, Paul D. Bieniasz, Benhur Lee, Naohiro Inohara, Irina Matei, Richard K. Plemper, Melody Y. Zeng

**Affiliations:** a Gale and Ira Drukier Institute for Children’s Health, Weill Cornell Medicine; New York, NY, USA; b Department of Pediatrics, Weill Cornell Medicine; New York, NY, United States of America; c Institute for Biomedical Sciences, Georgia State University; Atlanta, GA, United States of America; d Department of Medicine, Weill Cornell Medicine; New York, NY, United States of America; e Rogel Cancer Center, University of Michigan; Ann Arbor, MI, United States of America; f Laboratory of Retrovirology, The Rockefeller University; New York, NY, United States of America; g Jill Roberts Institute for Inflammatory Bowel Disease, Weill Cornell Medicine; New York, NY, United States of America; h Englander Institute for Precision Medicine, Weill Cornell Medicine; New York, NY, United States of America; i Department of Medicine, Division of Endocrinology, Diabetes, and Metabolism, University of Illinois at Chicago; Chicago, Illinois, United States of America; j Jesse Brown Veterans Affairs Medical Center; Chicago, Illinois, United States of America; k Howard Hughes Medical Institute, The Rockefeller University; New York, NY, United States of America; l Department of Microbiology, Icahn School of Medicine at Mount Sinai; New York, NY, United States of America

## Abstract

The gut microbiome is intricately coupled with immune regulation and metabolism, but its role in Coronavirus Disease 2019 (COVID-19) is not fully understood. Severe and fatal COVID-19 is characterized by poor anti-viral immunity and hypercoagulation, particularly in males. Here, we define multiple pathways by which the gut microbiome protects mammalian hosts from SARS-CoV-2 intranasal infection, both locally and systemically, via production of short-chain fatty acids (SCFAs). SCFAs reduced viral burdens in the airways and intestines by downregulating the SARS-CoV-2 entry receptor, angiotensin-converting enzyme 2 (ACE2), and enhancing adaptive immunity via GPR41 and 43 in male animals. We further identify a novel role for the gut microbiome in regulating systemic coagulation response by limiting megakaryocyte proliferation and platelet turnover via the Sh2b3-Mpl axis. Taken together, our findings have unraveled novel functions of SCFAs and fiber-fermenting gut bacteria to dampen viral entry and hypercoagulation and promote adaptive antiviral immunity.

## Introduction

Coronavirus disease 2019 (COVID-19), caused by the newly emerged coronavirus SARS-CoV-2, has created unprecedented global health and economic crises.^1^ Reduced vaccine efficacy in immunocompromised populations, such as patients with cancer or autoimmune diseases, further underscores the importance of continued research efforts to develop therapeutic modalities that complement vaccine-focused efforts to combat the COVID-19 pandemic. The human angiotensin-converting enzyme 2 (ACE2) mediates the entry of SARS-CoV-2 for infection and the manifestation of COVID-19.^2^ ACE2 is expressed throughout the body, including in the intestinal epithelial cells of the gastrointestinal (GI) tract.^3^ SARS-CoV-2 has frequently been detected in stool samples;^4^ GI symptoms are among the early symptoms in up to 50% of COVID-19 patients.^5,6^ Persistent intestinal reservoirs of SARS-CoV-2 might drive continued evolution of B cell memory responses in recovered or asymptomatic COVID-19 patients.^7^ Dysfunction of the coagulation response has emerged as another hallmark that contributes to the mortality and morbidity of COVID-19.^8–11^

The gut microbiome has been found to affect the outcome of other viral respiratory infections, such as influenza, as well as the development of chronic lung conditions such as asthma and COPD.^12,13^ Several recent studies have found evidence of altered gut microbiome composition in COVID-19 patients.^14^ However, the role of the gut microbiome in the susceptibility to SARS-CoV-2 infection and COVID-19 remains undefined.

In this study, we have demonstrated that short-chain fatty acids (SCFAs), derived from fiber-fermenting commensal bacteria, downregulate ACE2 and reduce infection by a VSV pseudovirus expressing SARS-CoV-2 spike in both mice and hamsters, promote antiviral T cell responses and the formation of spike-specific B and T cells, and dampen the coagulation response. Our findings potentially unravel gut microbiome-linked signaling pathways that can be harnessed in the development of preventive and therapeutic measures to mitigate infection by not only SARS-CoV-2 but other viral pathogens as well.

## Results

### Clostridia *species and SCFAs downregulate ACE2 expression in the intestine and lung*

**Fig 1.**
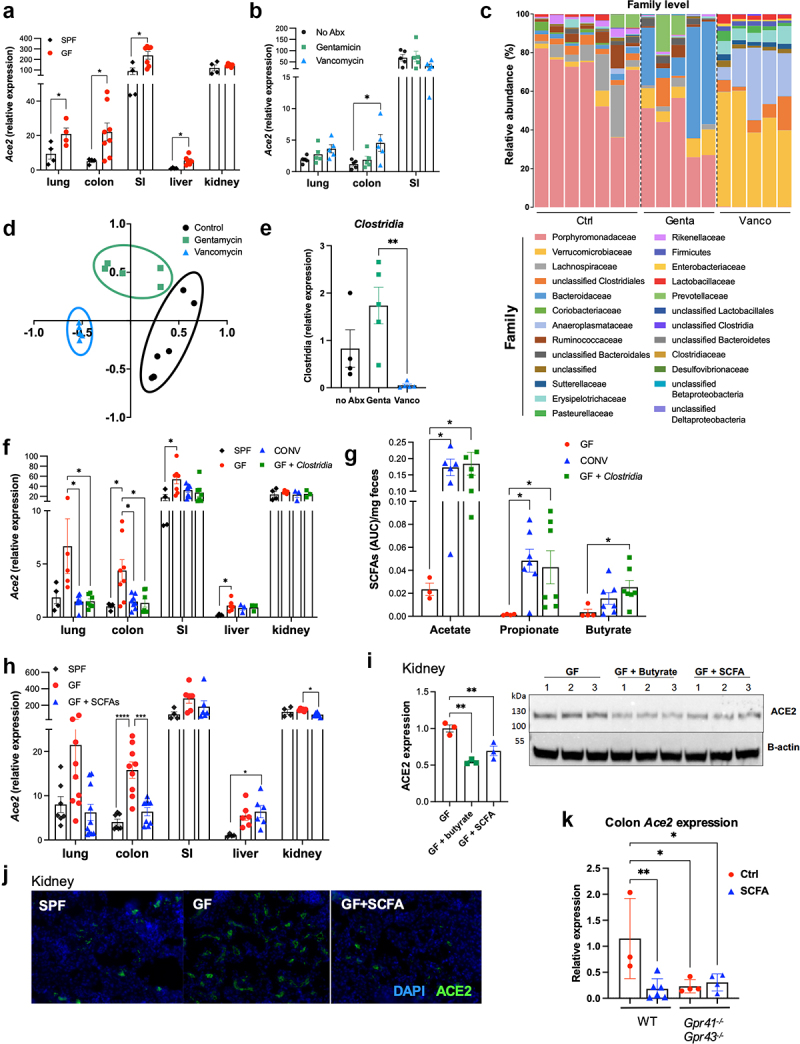
Commensal Clostridia species reduce Ace2 expression via SCFA production. (A) *Ace2* mRNA expression in male SPF and GF mice. (B) *Ace2* mRNA expression in SPF and GF mice treated with antibiotics for two weeks. (C–D) 16S rRNA sequencing was performed. (C) Relative abundance is shown at the family level; (D) non-metric multidimensional scaling analysis is shown. (E) The relative abundance of *Clostridia* species in fecal pellets of antibiotic-treated mice was measured by qPCR. (F–G) *Ace2* mRNA expression (F) and fecal SCFA concentrations (G) in SPF, GF, and GF mice colonized with either bulk fecal bacteria from SPF mice (CONV, conventionalized) or fecal bacteria enriched for *Clostridia*. (H) *Ace2* mRNA expression in SPF, GF, or SCFA-treated GF mice. (I–J) ACE2 protein expression was measured via Western blot (i; densitometry relative to the Ctrl condition) and immunofluorescence (j; images are representative of 2 independent experiments) in kidneys from GF mice given control or SCFA water (butyrate alone or a cocktail of acetate, butyrate, and propionate). (K) *Ace2* mRNA expression in the colons of control- or SCFA-treated wild type or *Gpr41*^−/−^*Gpr43*^−/−^ mice. Error bars indicate mean±SEM. Significance was determined using one-way ANOVA with Tukey’s test for multiple comparisons. (A–H) represent 2-3 independent experiments; images in (I–K) are representative of 2 independent experiments. SI = small intestine; FB = fecal bacteria. *p<0.05; **p<0.01. See also **Figures S1 and S2A.**

To first address whether *Ace2* expression is affected by the gut microbiome, we compared *Ace2* expression in age- and sex-matched C57BL/6 specific pathogen-free (SPF) and germ-free (GF) mice via qPCR. *Ace2* expression was elevated at the mRNA level in the lungs, colons, small intestines, and livers in GF mice (Figure 1A), suggesting a possible role for the gut microbiome in the regulation of *Ace2*. Flow cytometry analysis of kidney cells revealed higher protein expression of ACE2 in the kidneys of GF mice (**Figure S1A**) despite no significant difference at the mRNA level, but protein expression in the other tissues was too low for accurate quantification. The expression of *Tmprss2*, a transmembrane serine protease that primes the spike protein for viral entry,^2^ was not significantly altered (**Figure S1B**).

To identify gut bacteria that modify ACE2 expression, we first treated SPF mice with gentamicin or vancomycin in drinking water for 2 weeks. We observed increased *Ace2* expression in the colons of vancomycin-treated mice (Figure 1B). Both gentamicin and vancomycin treatments led to significant alterations of the gut microbiome and selective depletion of specific bacterial species (Figure 1C-D). Vancomycin is active against a diverse range of gram-positive and gram-negative bacteria, including the bacterial clusters Clostridium IV (in the family Ruminococcaceae) and Clostridium XIVa (in the family Lachnospiraceae), which are major producers of short-chain fatty acids (SCFAs).^15–17^ Accordingly, 16S rRNA sequencing of fecal DNA and qPCR quantification confirmed a near-total loss of *Clostridia* in vancomycin-treated mice (Figure 1C-E). To determine whether *Clostridia* specifically regulate *Ace2* expression, we colonized GF mice with fecal bacteria from SPF mice or fecal bacteria enriched by chloroform-resistant *Clostridia* as previously described^18^ (**Figure S1C**). While *Tmprss2* was not affected, *Ace2* expression was significantly reduced in the colons of GF mice colonized with fecal bacteria (CONV, conventionalized); importantly, colonization with *Clostridia-*enriched bacteria reduced *Ace2* expression to the levels seen in SPF mice (Figure 1F**, Figure S1D**).

*Clostridia* are major producers of SCFAs, such as acetate, butyrate, and propionate.^17^ Accordingly, fecal butyrate and propionate concentrations were drastically decreased in vancomycin-treated mice (**Figure S1E**). Likewise, colonization with SPF fecal bacteria or *Clostridia-*enriched bacteria in GF mice significantly increased fecal propionate and butyrate (Figure 1G). To determine if SCFAs alone could reduce *Ace2* expression, we administered a combination of three SCFAs (40mM acetate, 25.9mM propionate, and 67.5mM butyrate^19^) in drinking water to GF mice for 2 weeks, which reduced *Ace2* expression in the colons and kidneys, and reduced *Tmprss2* expression in the lung and liver (Figure 1H**, Figure S1F**). Similarly, western blot analysis (Figure 1I) and immunofluorescence imaging (Figure 1J) of kidneys from GF mice treated with the SCFA cocktail or butyrate alone showed reduced expression of ACE2 at the protein level. Interestingly, this phenotype was observed only in male but not female SCFA-treated mice (**Figure S1G**), suggesting a sex-biased mechanism behind SCFA regulation of ACE2 expression.

SCFAs directly interact with the G protein coupled receptors GPR41 (FFAR3) and GPR43 (FFAR2), which are expressed on diverse tissues including intestinal epithelia and immune cells.^20^ SCFA treatment did not affect *Ace2* expression in the lungs or colons of *Gpr41^−/−^Gpr43^−/−^, Gpr41^−/−^*, or *Gpr43^−/−^* mice (Figure 1K, **Figure S1H**), indicating that both receptors are likely involved in mediating SCFA regulation of *Ace2* expression. Additionally, the treatment of human liver organoids with SCFAs for 24 hours resulted in a reduction of *Ace2* expression (**Figure S2A**), suggesting that SCFAs may directly affect *Ace2* expression.

### SCFAs confer protection against intranasal infection in animals

**Fig 2.**
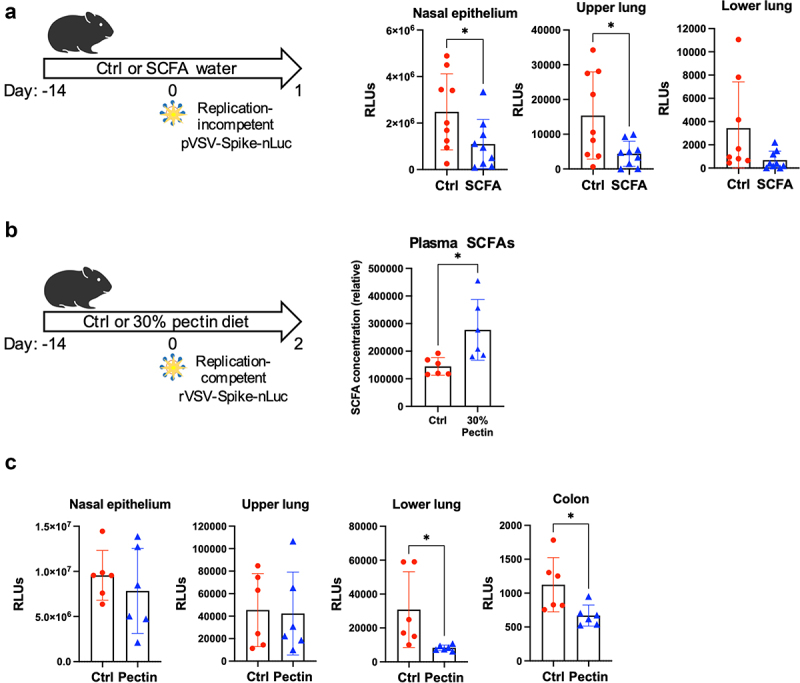
Treatment with SCFAs or SCFA-promoting pectin reduces viral entry in animals. (A) Luciferase activity in tissue homogenates from control or SCFA-treated Syrian hamsters 24 hours post infection with pVSV/Spike-nLuc pseudovirus. (B) Plasma SCFA concentrations from hamsters given a control diet or 30% pectin diet for two weeks were measured via LCMS; measurements for individual SCFAs are shown in **Figure S2C**. (C) Luciferase activity in tissue homogenates from control or pectin-treated Syrian hamsters 48 hours post infection with replication-competent rVSV/Spike-nLuc. Error bars indicate mean±SEM. Significance was determined using unpaired t-test. All data represent 2-3 independent experiments. RLU = relative light unit; SI = small intestine. *p<0.05. See also **Figure S2.**

Since ACE2 is the major entry receptor for SARS-CoV-2, we hypothesized that SCFA downregulation of ACE2 might reduce viral entry. Accordingly, we treated 9-week-old male Syrian hamsters with SCFA water for 2 weeks before intranasal infection with 5x10^5^ infectious units (IU) of a replication-incompetent vesicular stomatitis virus (VSV) pseudovirus expressing SARS-CoV-2 spike protein and nanoluciferase (designated **pVSV/Spike-nLuc**). After 24 hours, we analyzed viral burdens by measuring luciferase activity in homogenized tissues. Luciferase activity in the lung and nasal epithelium of SCFA-treated hamsters was reduced compared to that of control hamsters, indicating reduced infection (Figure 2A).

Dietary fibers, such as pectin, can be fermented by gut bacteria into SCFAs.^21^ We analyzed the expression of *Ace2* and *Tmprss2* in mice whose diets were supplemented with 5% or 30% pectin, and found both to be reduced in both the lungs and colons of mice receiving pectin supplementation (**Figure S2B**). We gave hamsters a 30% pectin diet for 2 weeks and found increased total SCFA levels in the plasma (Figure 2B, **Figure S2C**) and reduced luciferase activity in the lung and colon following infection with a replication competent chimeric VSV virus expressing SARS-CoV-2 spike and nanoluciferase (designated **rVSV/Spike-nLuc**; Figure 2C). Similar increases in plasma SCFA levels were observed in mice given 30% pectin for 2 weeks or hamsters given SCFA water for 2 weeks (**Figure S2D-E**). Of note, measurement of fecal SCFA levels proved unfeasible due to the high level of fat trapping in the stool of pectin-treated hamsters, which prevented efficient extraction of fecal metabolites for quantification. We additionally infected SCFA-treated GF mice with a replication-competent chimeric VSV virus expressing GFP and the SARS-CoV-2 beta variant spike protein (designated **rVSV/Spikeβ-GFP**), which can use murine ACE2 to infect cells.^22^ Consistent with our results in hamsters, 2 d post infection, we found reduced infection in the lungs of SCFA-treated GF mice, as determined by measuring GFP+ cells by flow cytometry (**Figure S2F**).

**Fig 3.**
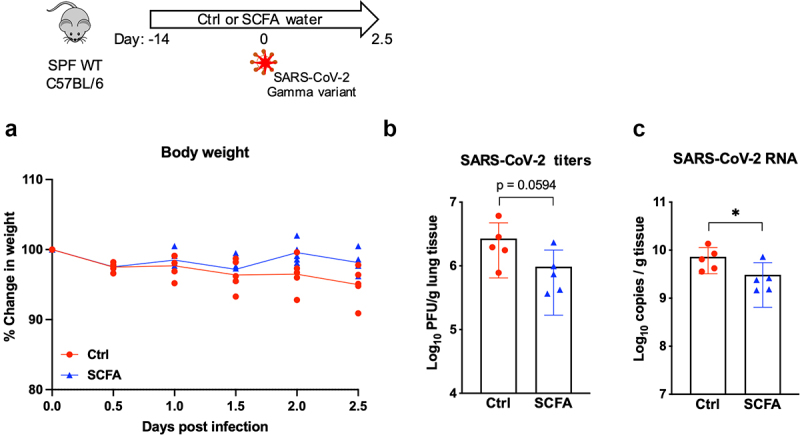
SCFA treatment reduces SARS-CoV-2 viral burdens in mice. Male SPF mice were treated with control or SCFA water for two weeks before infection with 4x10^5^ PFU of SARS-CoV-2 (gamma variant). (A) Weight change is shown as percentage of starting body weight. 2.5 d post infection, lung tissue was harvested and infectious viral titers (B) and viral RNA (C) were measured. Error bars indicate mean±SEM. For (A), significance was determined using two-way ANOVA with Šídák’s multiple comparisons test; for (B) and (C), significance was determined using unpaired t-test. *p<0.05

In order to confirm whether SCFA treatment is protective in the context of SARS-CoV-2 infection, we treated wild-type male SPF mice with SCFA water for 2 weeks before infection with 4x10^5^ PFU of SARS-CoV-2 gamma variant, and harvested lung tissue 2.5 d post infection, when lung viral titer peak. SCFA-treated mice exhibited a trend towards reduced weight loss (Figure 3A), as well as reduced viral titers (Figure 3B) and viral RNA (Figure 3C) in the lung. Together, these data indicate that SCFAs reduce viral burden following infection with SARS-CoV-2 or VSV-SARS-CoV-2 chimeras.

### SCFAs enhance adaptive immunity against VSV/SARS-CoV-2 chimeric viruses via GPR41/GPR43 in a sex-dependent manner

**Fig 4.**
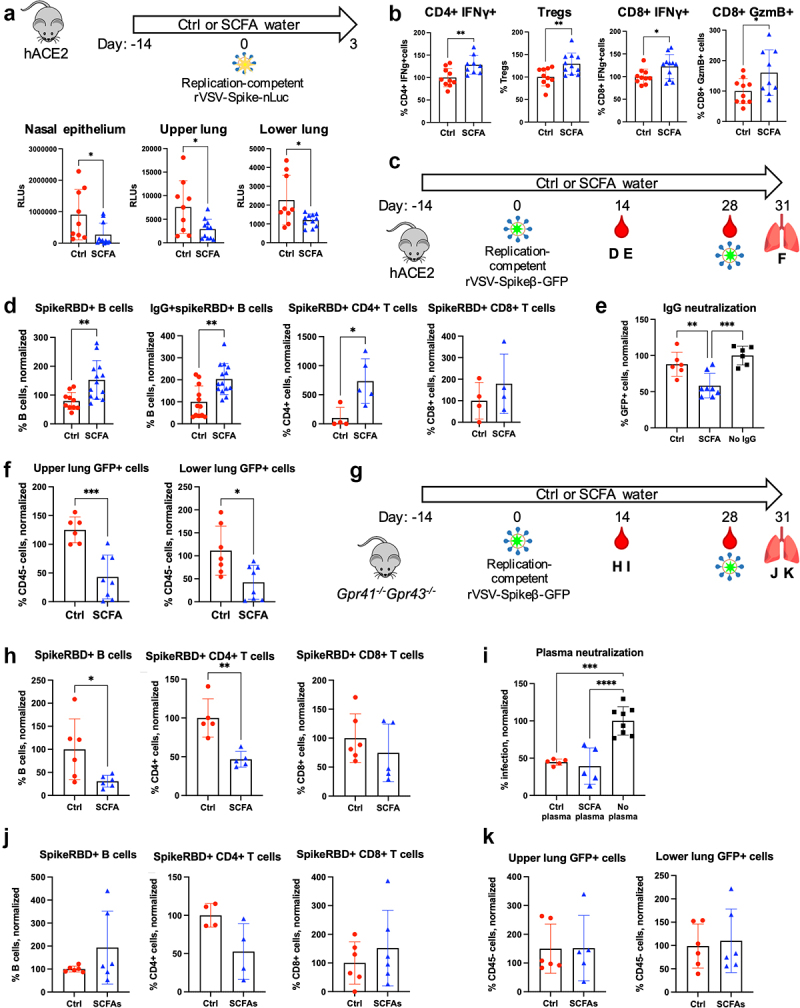
SCFAs enhance adaptive immunity against rVSV/Spikeβ-GFP via GPR41/GPR43 in a sex-dependent manner. (A–B) hACE2 mice were given control or SCFA water for two weeks before intranasal inoculation with replication-competent rVSV/Spike-nLuc. 48h later, (A) viral burdens were determined by measuring luciferase activity; and (B) lung T cells were stimulated with PMA/ionomycin for two hours before flow cytometry analysis. (C–K) The impact of SCFA treatment on adaptive immunity to rVSV/Spikeβ-GFP. (C,G) Overview of experiment. hACE2 (C) or *Gpr41*^−/−^*Gpr43*^−/−^ (G) mice were given control or SCFA water for two weeks before intranasal infection with 6x10^4^ IU of replication-competent rVSV/Spikeβ-GFP. Blood was collected two and four weeks post infection for analysis of immune cells. Four weeks post infection, mice were reinfected with 5x10^5^ IU of rVSV/Spikeβ-GFP and sacrificed after 3 d. (D–E, H–I) Two weeks following the primary infection, (D, H) blood immune cells were analyzed via flow cytometry, and purified IgG (E) or plasma (i) preincubated with rVSV/Spikeβ-GFP before infecting Vero-TMPRSS2 cells. 24 hours later, GFP+ cells were measured via flow cytometry. Results from only the male mice are shown. Data are shown as percentage of the no IgG/plasma condition. (F, J-K) Lung B and T cells (**J**) and GFP+ cells (F, K) in the lung 72 hours after the secondary infection. Error bars indicate mean±SEM. For (E) and (I), significance was determined using one-way ANOVA with Tukey’s test for multiple comparisons; for all other panels, significance was determined using unpaired t-test. All data represent 2 independent experiments. *p<0.05; **p<0.01; ***p<0.001; ****p<0.0001. See also **Figures S3-S4**.

SCFAs can prime the immune system to respond to infection.^13^ To examine the impact of SCFA treatment on the immune response to SARS-CoV-2 infection independently of its effect on ACE2 expression, we used mice expressing both mouse and human ACE2,^23^ designated **hACE2 mice**, in which human ACE2 is constitutively expressed in epithelial cells under the K18 promoter and is thus unaffected by SCFA treatment (**Figure S3A**). We treated hACE2 mice with SCFA water for 2 weeks before intranasal infection with rVSV/Spike-nLuc, which can interact with human ACE2 but not mouse ACE2. After 48 hours, we observed reduced luciferase activity in the nasal epithelium, lungs, and small intestines of SCFA-treated mice compared to control mice, indicating reduced infection (Figure 4A**, Figure S3B**). Flow cytometry analysis of lung immune cells revealed increased IFNγ expression in CD4+ and CD8+ T cells, increased Treg development, and heightened granzyme B production in CD8+ T cells (Figure 4C**, Figure S3C**). SCFA-treated mice also had more dendritic cells in the lung, although lung macrophages and polymorphonuclear cells (PMN) were not affected (**Figure S3D**). Together, these results suggest that SCFAs promote antiviral immune responses in the lung during acute infection.

To determine whether SCFAs affect the memory response, we treated hACE2 mice with control or SCFA water for 2 weeks before intranasal administration of 6x10^4^ PFU of rVSV/Spikeβ-GFP, followed by a secondary dose of 3x10^5^ PFU administered 4 weeks later (Figure 4C). Two weeks following the primary infection, flow cytometry analysis of blood immune cells showed that spike-specific B cells and CD4+ T cells were significantly elevated in the blood of SCFA-treated mice (Figure 4D). IgG from male SCFA-treated mice also more effectively neutralized rVSV/Spikeβ-GFP infection in Vero cells (Figure 4E); interestingly, this difference was not found in IgG from female mice (**Figure S4A**). Spike-specific B cells remained elevated for 4 weeks following the primary infection (**Figure S4B**). Following the secondary infection, GFP+ cells were significantly reduced in the lungs of SCFA-treated mice, indicating reduced viral burdens (Figure 4F). Importantly, SCFA treatment did not promote the generation of spike-specific B and T cells, antibody neutralization, or viral clearance in *Gpr41^−/−^Gpr43^−/−^* mice (Figure 4G-K), indicating a role for one or both of these receptors.

In hACE2 mice, human ACE2 expression is not affected by SCFA treatment (**Figure S3A**), which suggested that the effect of SCFA treatment on the immune response is independent of its effect on SARS-CoV-2 viral entry. To confirm this, we inoculated wild-type control or SCFA-treated mice with heat-inactivated rVSV/Spikeβ-GFP (two doses of 10^6^ PFU each, spaced 1 week apart; **Figure S4C**). Seven days following the second dose, SCFA-treated mice exhibited higher levels of spike-specific B cells and CD8+ T cells as well as increased IFNγ production in CD4+ T cells (**Figure S4D**). Plasma from SCFA-treated mice likewise neutralized rVSV/Spikeβ-GFP infection in Vero cells; consistent with our results in hACE2 mice, this phenotype was observed primarily in male mice (**Figure S4E**). Together, our data suggest that SCFAs enhance antiviral adaptive responses via the GPR41/GPR43 axis in a sex-biased manner.

### SCFAs regulate the coagulation response via the Sh2b3-Mpl axis to modulate platelet turnover

**Fig 5.**
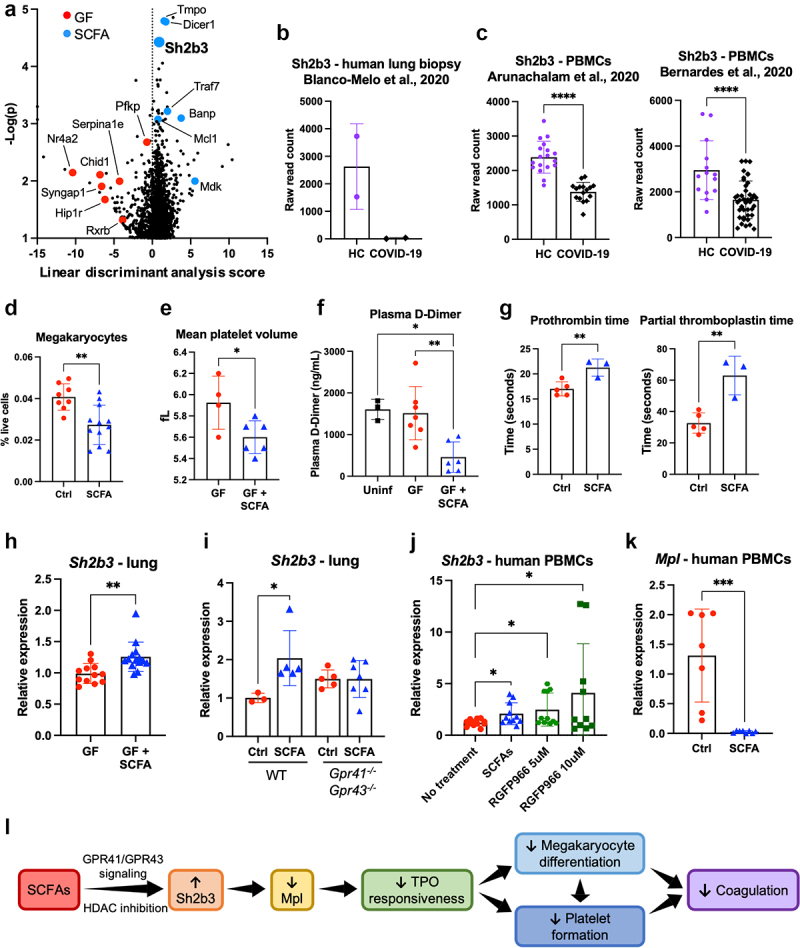
SCFAs regulate the coagulation response via the Sh2b3-Mpl axis to modulate platelet turnover. (A) RNA-seq analysis of lung tissue from control- or SCFA-treated GF mice. The volcano plot showing significantly differentially regulated genes is shown. Negative linear discriminant analysis (LDA) scores indicate genes that are upregulated in GF mice and positive LDA scores indicate genes that are upregulated in SCFA-treated mice. (B-C) Using publiclyavailable RNAseq datasets (GEO accession numbers: GSE147507, GSE152418, GSE161777), *Sh2b3* expression in human lung biopsy tissue (B) and human PBMCs (C) was compared between COVID-19 patients and healthy controls (HC). (D) Bone marrow megakaryocytes were measured in control- or SCFA-treated SPF mice via flow cytometry. (E) Mean platelet volume in control- or SCFA-treated GF mice. (F) Plasma D-dimer levels in control- or SCFA-treated GF mice 72 hours post infection with rVSV/Spikeb-GFP. (G) Prothrombin time and partial thromboplastin time assay on blood from male SPF mice given control water or SCFA water for two weeks. (H) Sh2b3 mRNA expression in control- or SCFA-treated GF mice. (I) Sh2b3 mRNA expression in control- or SCFA-treated SPF WT or *Gpr41-/-Gpr43-/-*mice. (J) *Sh2b3* mRNA expression in PBMCs from healthy human donors treated with SCFAs or the HDAC3 inhibitor RGFP966. (K) *Mpl* mRNA expression in PBMCs from healthy human donors treated with SCFAs. (L) Possible mechanism behind SCFA regulation of coagulation. Error bars indicate mean±SEM. For (F) and (I), significance was determined using one-way ANOVA with Tukey’s test for multiple comparisons; for all other panels, significance was determined using unpaired t-test. *p<0.05; **p<0.01; ***p<0.001; ****p<0.0001. See also Figure S5 and Table S1.

In order to identify other mechanisms by which SCFAs influence the outcome of SARS-CoV-2 infection, we performed RNA-seq on lungs from male GF mice given control or SCFA water for 2 weeks. Differentially regulated genes with *p* < 0.001 are shown in **Table S1**, and a volcano plot showing significantly altered genes is shown in Figure 5A. Of particular interest is the gene *Sh2b3*, also known as lymphocyte adaptor protein (LNK), which was upregulated in SCFA-treated mice (Figure 5A). *Sh2b3* is an adaptor protein that regulates a diverse array of signal transduction pathways, including the thrombopoietin (TPO) pathway; importantly, it is a major negative regulator of megakaryopoiesis and thrombopoiesis, thus playing an important role in regulating the coagulation response.^24^ Hypercoagulation and increased megakaryopoiesis are features of severe COVID-19.^9,11^ Using publicly available RNA-seq data,^25–27^ we found reduced expression of *Sh2b3* in a lung biopsy from a deceased COVID-19 patient compared to lung biopsies from two healthy controls (Figure 5B), as well as significantly reduced expression of *Sh2b3* in peripheral blood mononuclear cells (PBMCs) from hospitalized COVID-19 patients collected 5-20 d post-symptom onset (Figure 5C). We thus speculated that SCFA-mediated upregulation of *Sh2b3* might limit the coagulation response, potentially ameliorating COVID-19-associated coagulopathy.

Consistent with the established role of *Sh2b3* as a negative regulator of megakaryopoiesis, we found reduced megakaryocytes in the bone marrow of WT SPF SCFA-treated mice (Figure 5D). We performed a complete blood count on GF mice treated with control or SCFA water, and found that while SCFAs did not affect platelet count or platelet size distribution (**Figure S5A**), the mean platelet volume (MPV) was significantly reduced in SCFA-treated mice (Figure 5E). A higher MPV is indicative of a younger platelet population, composed of larger platelet cells that are more metabolically and thrombocytically active.^28^ The reduced MPV in SCFA-treated mice may therefore suggest reduced platelet turnover. Finally, we treated GF mice with SCFA water for 2 weeks before infecting with 6x10^4^ IU of rVSV/Spikeβ-GFP. Three days post infection, blood from SCFA-treated mice had significantly lower levels of D-dimer (Figure 5F) and elevated prothrombin time and partial thromboplastin time (Figure 5G), indicating a reduced coagulation response.

We confirmed our RNA-seq results by measuring *Sh2b3* expression via qPCR in lung tissue from SPF or GF mice given control or SCFA water for 2 weeks. Consistent with the RNA-seq results, we found that SCFA treatment significantly increased *Sh2b3* expression in the lungs (Figure 5H). However, *Sh2b3* expression was not affected by SCFA treatment in *Gpr41^−/−^Gpr43^−/−^, Gpr41^−/−^*, or *Gpr43^−/−^*,mice (Figure 5I**, Figure S5B**); likewise, SCFA treatment did not affect MPV, plasma D-dimer levels, or bone marrow megakaryocytes (**Figure S5C**) in *Gpr41^−/−^Gpr43^−/−^* mice. Since SCFAs are well-established inhibitors of histone deacetylases (HDACs),^20^and there is evidence that *Sh2b3* expression is sensitive to acetylation,^29,30^ we treated human PBMCs from healthy donors with SCFAs or the HDAC3 inhibitor RGFP966 and found that both were capable of promoting *Sh2b3* expression (Figure 5J), suggesting that SCFA regulation of Sh2b3 may have an epigenetic component in addition to the GPR41/GPR43 axis. Interestingly, unlike the ACE2 and antibody neutralization phenotypes we observed, SCFA enhancement of Sh2b3 did not exhibit a sex bias in either human PBMCs or mouse lungs (**Figure S5D-E**).

TPO signaling via its receptor, *Mpl* (myeloproliferative leukemia protein), which is primarily expressed in hematopoietic cells, drives megakaryopoiesis and is negatively regulated by *Sh2b3*.^24,31^ Since the liver is the primary producer of TPO, we investigated the expression of *Sh2b3* and TPO in the livers of control- or SCFA-treated GF mice, and found that while *Sh2b3* was upregulated following SCFA treatment, TPO expression was unaffected (**Figure S5F)**. However, we found that in human PBMCs, SCFA treatment significantly reduced *Mpl* expression (Figure 5K). Our data suggest that SCFA-mediated upregulation of *Sh2b3* leads to inhibition of *Mpl* signaling in megakaryocytes, reducing both megakaryocyte proliferation and platelet production, leading to reduced platelet turnover, and limiting coagulation (Figure 5L).

## Discussion

Altogether, we have elucidated a novel role for the gut microbiome, via SCFA production, in the host response to SARS-CoV-2 infection. SCFA treatment reduced the expression of *Ace2,* lowered viral burdens, and promoted adaptive immunity against VSV/SARS-CoV-2 chimeras. We have also uncovered a novel function of SCFAs in limiting the coagulation response via the *Sh2b3-Mpl* axis to modulate megakaryopoiesis and platelet turnover.

Our work suggests a gut-lung axis during SARS-CoV-2 infection that may dictate disease severity by regulating viral entry, adaptive immunity, and coagulation response. In the context of COVID-19, several studies have found altered microbiomes following SARS-CoV-2 infection.^32–36^ However, due to the high variability of the human microbiomes, which may have been influenced by the infection and medications in COVID-19 patients, controlled animal studies might help define the role of the gut microbiome in host response to SARS-CoV-2 infection. Interferon signaling is efficiently suppressed by at least 13 components of the SARS-CoV-2 genome, including spike protein, the nucleocapsid proteins nsp1 and nsp6, and accessory proteins ORF3b and ORF6.^37^ Poor T cell responses, characterized by low numbers of IFNγ+ CD4+ T cells and cytotoxic SARS-CoV-2-specific CD8+ T cells, are associated with severe or fatal COVID-19 outcomes.^38^ Enhanced megakaryopoiesis and excessive influx of neutrophils into the lung are further hallmarks of severe COVID-19.^8,26^ SCFAs modulate the immune response in many ways, including promoting regulatory and effector T cell development, T cell memory, and plasma cell differentiation.^13,39–41^ Here, we found that SCFAs enhance antiviral T cell responses, spike-specific B and T cells, and antibody neutralization following intranasal infection with a VSV pseudovirus expressing the spike protein. Importantly, we observed these phenotypes in hACE2 mice, in which human *Ace2* expression is not affected by SCFA treatment, and in wild-type mice inoculated with heat-inactivated virus, suggesting that the enhancement of immune responses that we observed is independent of the effect of SCFAs on ACE2-mediated viral entry, and may thus have an effect on other disease states as well.

We found that SCFAs affected *Ace2* expression and antibody neutralization only in male mice, but found no sex differences in the effect of SCFAs on the coagulation response. Our observations of a sex-biased effect of SCFA on antibody neutralization are intriguing. Females tend to have increased antibody responses to infection or vaccination,^42^ which has been reported in COVID-19 as well.^43–45^ In mice, the microbiome has sex-specific effects on antibody production and autoimmunity,^42^ and X inactivation escape of TLR7 has been linked to increased B cell responsiveness and class switching to IgG.^46^ Likewise, the mechanisms behind sex-specific regulation of *Ace2* are not fully explored, but *Ace2* expression is influenced by both Sry and 17β-estradiol, and *Ace2* has been found to escape X chromosome inactivation.^47–49^ The contributions of sex hormones and chromosomal sex on the interactions between the immune system and the microbiome have yet to be fully explored but may contribute to the sex bias seen in COVID-19 severity. Future work is required to delineate the mechanism underlying SCFA-mediated sex bias in adaptive immunity and to address whether this phenomenon is restricted to SARS-CoV-2 infection.

Our findings have uncovered a novel function of the gut microbiome in the regulation of coagulation. Suppression of the coagulation response via regulation of the Sh2b3-Mpl axis presents a third potential axis by which SCFAs might promote better SARS-CoV-2 infection outcomes. Dysfunction of the coagulation response has emerged as a hallmark of severe COVID-19,^9,11^ driven in part by hyperinflammation;^9^ this is also found in other severe viral pneumonias, including SARS, MERS, and H1N1 influenza,^50,51^ and accounts for a significant percentage of mortality and morbidity in cancer patients.^52^ Abnormalities associated with COVID-19 include capillary microthrombi, elevated D-dimer, venous thromboembolism, elevated risk of stroke, enhanced megakaryocyte activation, and thrombocytopenia often followed by rebound thrombocytosis.^9,11,26,50^ The latter, together with observations of elevated mean platelet volume and increased platelet activity in COVID-19 patients,^11^ suggest that COVID-19-associated hypercoagulation is partly due to increased platelet production in response to increased platelet consumption, resulting in a population of newer, more active platelets. It remains unclear why certain patients are more susceptible to hypercoagulation. Our findings suggest that the abundance of fiber-fermenting gut bacteria or SCFAs may influence the propensity for overexuberant coagulation in response to infection, by limiting platelet turnover through regulation of megakaryocyte responsiveness to TPO. The SCFA-Sh2b3-Mpl axis might be a potential therapeutic target to dampen hypercoagulation triggered by severe viral infections or other conditions.

Because SARS-CoV-2 requires BSL-3 conditions, the majority of experiments in our study were done using recombinant VSV-based pseudoviruses and chimeric viruses, which presents a major limitation. While these constructs enabled us to specifically examine the effect of SCFAs on viral entry and on immune responses to the SARS-CoV-2 spike protein, they may not fully recapitulate the complex interactions between SARS-CoV-2, the immune system, and the coagulation response. Our observations that SCFA treatment can reduce viral RNA levels following SARS-CoV-2 infection are encouraging, but more work is required to further examine the effect of SCFAs on the immune and coagulation responses in the context of a genuine SARS-CoV-2 infection.

Additional future work is also required to further elucidate the mechanisms underlying SCFA modulation of *Ace2* and *Sh2b3* expression. Our observations indicate that GPR41 and GPR43 are necessary for SCFA-mediated suppression of *Ace2* and enhancement of immune responses and *Sh2b3* expression; however, an epigenetic component also seems likely, as SCFAs are known histone deacetylase (HDAC) inhibitors.^20^ *Ace2* is upregulated by the class III HDAC SIRT1,^53^ and the HDAC inhibitor valproic acid (an SCFA derivative) reduces *Ace2* expression *in vitro*.^54^ The epigenetic regulation of *Sh2b3* is less well established, but there is evidence that *Sh2b3* expression is sensitive to acetylation,^29,30^ further supported by our study. However, more work is required to determine whether HDAC inhibition affects *Ace2* or *Sh2b3* expression *in vivo* and to define the relative contributions of the GPR41/GPR43 axis and the epigenetic axis. Further exploration of the mechanism by which SCFAs mediate *Ace2* and *Sh2b3* expression may lead to novel avenues for treatment not only of SARS-CoV-2 but a variety of disease states ranging from viral pneumonias to hypertension to stroke.

## Materials and Methods

### Animals

Wild-type or K18-hACE2 mice on C57BL/6 background, raised in germ-free or specific pathogen-free conditions, were originally purchased from the Jackson Laboratory and maintained and expanded in-house by the Zeng laboratory. *Ffar2*^−/−^ and *Ffar3^−/–^* mice on C57BL/6J background were generously provided by Dr Brian Layden (University of Illinois) and maintained and expanded in-house. *Ffar2^−/−^Ffar3^−/−^* mice were generated by crossing *Ffar2*^−/−^ and *Ffar3^−/–^* mice, and genotypes were confirmed by PCR. Syrian hamsters were purchased from Charles River Laboratories. All mice were 8 weeks of age, and all hamsters were 9 weeks of age at the time treatment commenced. Male mice were used except where otherwise specified. Littermate controls were used, and animals were cohoused after weaning. SPF animals were housed in microisolator cages in the barrier facility of Weill Cornell Medicine. Infected animals were housed in the BSL2+ biocontainment facility of Weill Cornell Medicine or the BSL3 biocontainment facility at Georgia State University. Germ-free mice were bred and maintained in semi-rigid gnotobiotic isolators and transferred into individually ventilated isocages for experimentation. All animal experiments were approved by the Institutional Animal Care and Use Committee (IACUC) at Weill Cornell Medicine (IACUC protocol 2019-0004) or Georgia State University (IACUC protocols A20043 and A20044).

### Cell lines

Vero-TMPRSS2 cells were generously provided by Dr Benhur Lee (Icahn School of Medicine at Mount Sinai) and are previously described.^55^ Vero-TMPRSS2 cells were maintained in DMEM (Gibco 11965-092) supplemented with 10% fetal bovine serum (FBS; Gibco 10437-028) and penicillin–streptomycin (1:50; Sigma P4458) at 37°C in a 5% CO_2_ atmosphere. I1 hybridoma cells (ATCC CRL-2700) were obtained from the ATCC and maintained at 37°C in 100% room air, in minimal essential media with Hank’s balanced salts (Gibco 11575032) supplemented with 2mM L-glutamine (Sigma G7513) and 20% FBS.

### Generation of rVSVΔG/NG/NanoLuc-SARS-CoV-2 pseudovirus (pVSV/Spike-nLuc)

The rVSVΔG/NG/NanoLuc-SARS-CoV-2 pseudovirus was generated as previously described.^56^ Briefly, an mNeonGreen/FMDV2A/NanoLuc luciferase cDNA cassette was inserted into the rVSVΔG plasmid (Kerafast EH1007)^57^ between the M and L genes (maintaining the VSV intergenic sequences). The resulting plasmid was then transfected into 293T cells infected with recombinant T7-expressing vaccinia virus (vTF7-3), together with the rVSV pseudovirus rescue plasmids (pBS-N, pBS-P, pBS-L, and pBS-G; Kerafast EH1008), in order to rescue VSV-G-expressing pseudovirus particles carrying the NeonGreen/NanoLuc reporter cassette. Twenty-four hours post transfection, the supernatant was collected, filtered using a 0.1 μm filter to remove the residual vaccinia virus, and used to infect 293T cells transfected with SARS-CoV-2 spike protein lacking the C-terminal 19 codons. After 16 hours, the supernatant was collected, centrifuged briefly to remove cell debris, and concentrated via PEG precipitation. The concentrated pseudovirus was aliquoted and stored at -80°C. Prior to infection, pseudovirus stocks were incubated for 1 hour at 37°C with 20% I1 hybridoma (ATCC CRL-2700) supernatant, which contains anti-VSV-G antibodies, to neutralize residual VSV-G pseudovirus particles.

### Generation of replication-competent VSV/SARS-CoV-2 chimera (rVSV/Spike-nLuc)

The replication-competent VSV/SARS-CoV-2 chimera was generated as previously described.^56^ Briefly, a codon-optimized sequence encoding the SARS-CoV-2 spike protein (SinoBiological VG40592-UT) lacking the C-terminal 18 codons was inserted into a plasmid encoding the VSV antigenome containing Nano luciferase, immediately upstream of the L protein, following a strategy previously described for the generation of a VSV/HIV-1 chimera.^58^ The resulting plasmid was then transfected into vTF7-3-infected 293T cells, together with the rVSV rescue plasmids, in the same manner as described for the generation of VSVΔG pseudovirus. The supernatant was filtered to remove residual vaccinia virus, passaged once in 293T cells transfected with VSV-G protein to amplify, and then subsequently passaged several times in ACE2-expressing 293T cells.

### Generation of replication-competent rVSV/Spikeβ-GFP chimera

The replication competent chimeric rVSV-Spikeβ-GFP virus was generated as previously described.^59^ Briefly, the pEMC-VSV-eGFP-CoV2-S plasmid (GenBank Accession: MW816496) was generated by cloning the VSV-eGFP sequence^60^ into pEMC vector (pEMC-VSV-eGFP) and then replacing the VSV-G open reading frame with a codon-optimized SARS-CoV-2 Spike (BEI NR-52310) carrying the beta variant mutations D80A, D215G, del242-244, K417N, E484K, N501Y, D614G, and A701V (from EPI_ISL_745109; GenBank Accession: MW816499), and truncated to lack the final 21 amino acids.^61^ Sequences encoding the VSV N, P, M, G, and L proteins were also cloned into pCI vector to make expression plasmids for virus rescue, resulting in plasmids: pCI-VSV-N, pCI-VSV-P, pCI-VSV-M, pCI-VSV-G, and pCI-VSV-L. To rescue the virus, 293T cells expressing ACE2 and TMPRSS2 were transfected with 2000 ng of pEMC-VSV-EGFP-CoV2 spike, 2500 ng of pCAGGS-T7opt66, 850 ng of pCI-VSV-N, 400 ng of pCI-VSV-P, 100 ng of pCI-VSV-M, 100 ng of pCI- VSV-G, 100 ng of pCI-VSV-L using Lipofectamine LTX (Invitrogen). Cells were maintained for 4–5 d until GFP-positive syncytia appeared. Rescued viruses were then amplified in Vero-CCL81-TMPRSS2 cells^55^ and concentrated via PEG precipitation.

### Human PBMCs

Blood samples were obtained from healthy adult volunteers (seven females and three males) and PBMCs were extracted for *in vitro* SCFA or HDAC inhibitor treatment. Informed consent was obtained from all subjects. This study was approved by the Weill Cornell Medicine Institutional Review Board (protocol # 0604008488).

### Oral administration of antibiotics, SCFAs, or pectin

Antibiotic drinking water was prepared using 1 g gentamicin sulfate (Millipore 345814) or 0.5 g vancomycin hydrochloride (Sigma V2002) in 1 L tap water and filtered through a 0.22 μm sterile filter. Mice were given antibiotic drinking water ad libitum for 3 weeks before tissues or fecal pellets were collected for further analyses. SCFA drinking water was prepared following a previously described protocol,^19^ using 67.5 mM sodium butyrate (Sigma 303410), 40 mM sodium acetate (Sigma S2889), and 25.9 mM sodium propionate (Sigma P1880) in 1 L tap water and filtered through a 0.22 μm sterile filter. Mice and hamsters were given SCFAs in drinking water ad libitum for a minimum of 2 weeks before further experimentation. For pectin diet experiments, mice or hamsters were given control diet, 5% pectin diet, or 30% pectin diet ad libitum for a minimum of 2 weeks before further experimentation. The 5% and 30% pectin diets were custom designed by Research Diets based on the standard AIN93G formula (Research Diets D10012G). The formulation for each diet is listed in **Table S2**.

### qPCR

Total RNA was extracted from tissue samples using Trizol (Invitrogen 15596026) following the manufacturer’s instructions. 2000ng RNA per sample was used to generate cDNA using Applied Biosystems High-Capacity cDNA Reverse Transcription Kit (Applied Biosystems 4368814) following the manufacturer’s instructions. cDNA was diluted to 1:5 in water before performing qPCR using Intact Genomics ig SYBR Green 2x Master Mix (Intact Genomics 3354). qPCR was carried out with the CFX384 Real-Time System C1000 Touch Thermal Cycler (Bio-Rad Laboratories). Cycling conditions were as follows: Initial denaturation 95°C for 2 minutes, 40 cycles of denaturation at 95°C for 5 seconds followed by annealing/extension at 60°C for 30 seconds. Primers used are listed in **Table S3**. Relative expression was calculated using the ΔΔCt method, using β-actin as the reference gene.

### 16S rRNA sequencing analysis

Fecal samples were freshly collected, and DNA was extracted using the E.Z.N.A. stool DNA kit (Omega Bio-tek D4015-02). The V4 region of the 16S rRNA gene was amplified using universal primers (listed in **Table S3**) and sequenced using an Illumina MiSeq apparatus as previously described.^62^ Paired-end reads were analyzed and classified into operational taxonomic units (OTUs) at >97% identity level using Mothur v.1.40.5.^62,63^ Taxonomic assignments were determined using the SILVA 16S rRNA reference file release 132^64^ and the Ribosomal Database Project (RDP) training set version 16.^65^

## *Preparation of* Clostridia*-enriched fecal bacteria*

*Clostridia*-enriched fecal bacteria were prepared as previously described.^18^ Briefly, a donor SPF WT mouse was euthanized, and the entire gut from rectum to duodenum was removed intact into a petri dish in the laminar flow hood. This was immediately introduced into an anaerobic chamber. One fecal pellet from the upper colon and a similar amount of cecal contents were removed and homogenized into 18 mL anaerobic PBS. 15 mL was transferred to a Hungate tube, sealed, and removed from the chamber. 470 µL chloroform was injected through the rubber stopper, and the contents were shaken vigorously by hand for 5 minutes and then in a rotary shaker for 1 hour at 37°C. To remove the chloroform, a 25G exhaust needle was inserted through the rubber stopper, and then a 5” × 25G pencil point needle (Reli) was carefully inserted until reaching the bottom of the tube. This was connected to a CO2 tank via a regulator and 0.2 µm filter. CO_2_ was bubbled through the solution for 10 minutes, and the solution was taken immediately for gavage into GF mice (0.2 mL per mouse).

### Quantification of SCFAs in mouse fecal samples

After 2 weeks of antibiotic treatment or colonization with fecal bacteria, fecal pellets were collected, and approximately 10 mg of each sample was directly resuspended in 100 µL SCFA derivatization solution (prepared as previously described^66^). Glass beads were added, and samples were vortexed for 10 minutes to disperse the feces. Samples were then incubated at 60°C for 1 hour and chilled on ice and then centrifuged at 21000 × g for 20 minutes. The supernatant was then mixed with the D3-leucine internal standard (Sigma 486825) at a ratio of 5 parts supernatant to 1 part internal standard solution (0.0125 mg/mL D3-leucine). Samples were analyzed by LC-MS using the following solvent system for SCFA detection: A: H_2_O with 0.1% formic acid (Sigma F0507); B: Methanol (Sigma 322415) with 0.1% formic acid. 1 μL of each sample was injected, and the flow rate was 0.35 mL/min with a column temperature of 40 C. The gradient for HPLC-MS analysis was as follows: 0-6.0 min 99.5% A – 70.0% A, 6.0-9.0 min 70.0% A-2.0%A, 9.0-9.4 min 2.0%A-2.0%A, 9.4–9.6 min 2.0%A 99.5% A. Peaks were assigned by comparison with authentic standards, and relative analyte concentrations were quantified by comparing their peak areas with those of the internal standard (D3-Leucine).

### Western blotting of ACE2

Tissues were homogenized in RIPA buffer with protease and phosphatase inhibitors (Roche 11873580001), and tissue lysates were collected. Protein concentrations in the lysates were measured using a Pierce™ BCA Protein Assay Kit (Thermo Scientific 23225). Lysates were normalized and then resolved using NuPAGE 4–12% Bis-Tris gels (Invitrogen NP0321) and transferred onto a PVDF membrane (Bio-Rad Laboratories). The membrane was blocked using 3% non-fat milk for 1 h at room temperature and then probed with a rat anti-mouse ACE2 antibody (1:500; R&D Systems MAB3437) at 4°C overnight. The membrane was probed with goat anti-rat HRP antibody (1:5000; Invitrogen 31470) for 1 h at room temperature. SuperSignal™ West Pico PLUS Chemiluminescent Substrate (Thermo Scientific 34579) was added to the membrane, and the blot was imaged using a ChemiDoc™ XRS+ Molecular Imager (Bio-Rad Laboratories). β-actin antibody (GenScript A00730) was used as a loading control. Densitometry analyses of protein bands were performed using ImageJ software.

### Immunofluorescence staining of ACE2 in mouse tissues

Mouse tissues were fixed using 4% paraformaldehyde (PFA; Thermo Scientific J19943-K2) overnight. Tissues were washed with PBS and then placed into 30% sucrose overnight. Tissues were then placed into Tissue-Tek O.C.T. gel (Sakura 4583), and 5 μM sections were placed onto microscope slides. Tissue sections were permeabilized with 0.5% TritonX100 for 15 min at room temperature. Antigen retrieval was conducted on tissues using Citrate Buffer Antigen Retriever (Sigma C9999) for 30 min at 85°C. After a wash step, sections were blocked with 5% horse serum in PBS for 60 min at room temperature, then incubated with goat anti-mouse ACE2 (1:200; R&D Systems AF3437) at 4°C overnight. After a wash step, sections were incubated with donkey anti-goat IgG H&L Alexa Fluor 488 antibody (1:200; Abcam ab150129) and counterstained with DAPI (1:5000) for 5 min. After staining, coverslips were mounted onto tissue sections using ProLong™ Gold Antifade Mountant (Invitrogen P36930). The images were captured using a Zeiss AxioObserver inverted fluorescence microscope.

### Human liver organoid experiments

Human liver organoids were generated as previously described.^67^ Briefly, human hepatocytes and human nonparenchymal fractions were obtained from the Liver Tissue Cell Distribution System (Pittsburgh, Pennsylvania). Human liver tissue was digested to obtain cellular fractions. 2,500 cells were mixed 1:1 with Matrigel (Corning 354234) and placed at the bottom of a 6-well plate at a density of 5 drops per well. Plates were incubated for 5 min at 37°C before culture media was added. Organoid culture media consisted of DMEM/F12 media (Thermo Fisher 10565018) supplemented with B27 supplement without vitamin A (1:50; Life Technologies 12587-010), N2 supplement (1:100; Life Technologies 17502-048), 1 mM N-acetylcysteine (Sigma A0737), 10% (vol/vol) Rspo1-conditioned medium (produced as previously described^68^), 10 mM nicotinamide (Sigma N0636), 10 nM recombinant human [Leu^15^]-gastrin I (Sigma G9145), 50 ng/mL recombinant human EGF (Peprotech AF-100-15), 100 ng/mL recombinant human FGF-10 (Peprotech 100-26), 25 ng/mL recombinant human HGF (Peprotech 100-39), 10 μM Forskolin (Tocris Bioscience 1099), and 5 μM A83-01 (Tocris Bioscience 2939). Organoids were maintained at 37°C in a 5% CO_2_ atmosphere, and the medium was changed every 3–4 d; cultures were initially split after 14 d and subsequently split every 5–7 d. SCFA treatment was initiated when the organoids were approximately 30% confluent (~2 d after splitting) by adding a cocktail of acetate, butyrate, and propionate to the organoid medium (low dose: 100µM acetate, 50µM butyrate, 200µM propionate; high dose: 1mM acetate, 0.5mM butyrate, 2mM propionate). After 48 hours, the growth medium was removed, and the organoids and Matrigel were homogenized in Trizol by pipetting up and down, after which RNA was extracted and qPCR performed as described above.

### Infection of mice and hamsters with pseudoviruses and chimeric viruses

Eight-week-old mice were treated with SCFAs for 2 weeks before intranasal infection with 6x10^4^ IU (20 µL per mouse, 10 µL per nare) of either the pVSV/Spike-nLuc pseudovirus, the replication-competent rVSV/Spike-nLuc chimera, or the replication-competent rVSV-Spikeβ-GFP chimera. Mice were euthanized via CO_2_ inhalation for 2 d (for pVSV/Spike-nLuc infection) or 3 d (for rVSV/Spike-nLuc or rVSV/Spikeβ-GFP infection) post infection. Nine-week-old male Syrian hamsters were given either SCFA drinking water or a high-fiber diet (AIN93G supplemented with 30% pectin) for 2 weeks before intranasal inoculation with 5x10^5^ IU (100 µL per hamster, 50 µL per nare) of either the pVSV-Spike-nLuc pseudovirus or the rVSV-Spike-nLuc chimeric virus. Hamsters were euthanized by CO_2_ inhalation 24 hours (for pseudovirus-infected hamsters) or 48 hours (for chimera virus-infected hamsters) post infection. For inoculation with heat-inactivated virus, rVSV/Spikeβ-GFP was incubated for 30 minutes at 56°C prior to inoculation.

### Measurement of viral loads

For animals infected with luciferase reporter viruses, infection was measured via luciferase production in infected tissues using the Promega Nano-Glo Luciferase Assay System (Promega N1120). Tissue samples were weighed, and 10 µL lysis buffer (PBS + 1% Tween-20) was added per 1 mg of tissue. Samples were minced with sterile scissors and sonicated briefly, then incubated at room temperature for a minimum of 10 minutes to allow lysis to proceed. 50 µL of tissue lysate was mixed with 100 µL Nano-Glo Luciferase Assay Reagent, and the total endpoint luminescence was read immediately. For animals infected with GFP reporter viruses, infection was measured by quantifying GFP+ cells via flow cytometry. Lung tissue was pushed through a 100 μm filter to create a single-cell suspension in PBS with 1% bovine serum albumin (BSA; Sigma A2153) and 0.1 mg/mL DNase I (Sigma DN25), then incubated with Fixable Viability Dye eFluor™ 780 (1:3000; eBioscience 65-0865-14) and Alexa Fluor 700 conjugated anti-CD45 antibody (1:200, BioLegend 103128) at 4°C for 30 minutes, washed and resuspended in PBS with BSA and DNAse I, and analyzed on a Cytek Aurora flow cytometer (BD).

### SARS-CoV-2 infections

Five-week-old male C57BL/6 mice were randomly divided into two study groups (n = 5 each) that received filtered tap or SCFA water (3.3 g sodium acetate; 7.5 g sodium butyrate and 2.5 g sodium propionate per 1 L water), respectively, during a 14-d pre-infection period. Animals were monitored daily. For infection, mice were transferred into ABSL-3 containment and inoculated intranasally with 4x10^5^ PFU (25 µl per nare) of SARS-CoV-2 gamma variant (lineage P.1., isolate hCoV-19/Japan/TY7-503/2021; BEI Resources NR54982). Infected animals were continued on filtered tap or SCFA water as before. Body temperature was monitored daily and body weight measured twice daily. All animals were euthanized 2.5 d after infection and lungs, colons and kidneys harvested. Lungs were weighed and homogenized with a bead beater in 300 µl PBS in 3 bursts of 30 seconds each at 4°C. Samples were clarified for 10 minutes at 4°C and 14,000 g, supernatants aliquoted and stored at -80°C prior to titration by plaque assays on Vero-TMPRSS2 cells. Virus titers were normalized to the weight of the input lung tissue and buffer volume. For qPCR-based detection of viral RNA, lung, colon, and kidney samples were stored in RNAlater at -80°C. Tissues were weighted and homogenized as above, followed by total RNA extraction using a RNeasy mini kit (Qiagen). All experimentation with SARS-CoV-2 was performed under ABSL3 biocontainment conditions and was approved by the Institutional Biosafety Committee (IBC) of Georgia State University (IBC protocol B20006).

### Plaque assay

Samples were serially diluted (tenfold dilutions starting at a 1:10 initial dilution) in DMEM medium supplemented with 2% FBS containing Antibiotic-Antimycotic (Gibco). The serial dilutions were added to Vero-TMPRSS2 cells seeded in 12-well plates at 3 × 10^5^ cells per well 24 hours previously. The virus was allowed to adsorb for 1 hour at 37 C. Subsequently, the inoculum was removed, and the cells were overlaid with 1.2% Avicel (FMC BioPolymer) in DMEM and incubated for 3 d at 37°C with 5% CO2. After 3 d, the Avicel was removed, cells were washed once with PBS, fixed with 10% neutral buffered formalin, and plaques were visualized using 1% crystal violet.

### Quantitation of SARS-CoV-2 viral RNA

SARS-CoV-2 RNA was detected using the nCoV_IP2 primer-probe set (National Reference Center for Respiratory Viruses, Institute Pasteur;^69^ see **Table S3**) which targets the SARS-CoV-2 RdRP gene. RT-qPCR reactions were performed using an Applied Biosystems 7500 real-time PCR system using the StepOnePlus real-time PCR package. For amplification, a TaqMan fast virus 1-step master mix (Thermo Fisher Scientific) was used. To determine RNA copies, raw data were processed using a standard curve that was created generated from viral cDNA using the nCoV_IP2 forward primer and the nCoV_IP4 reverse primer. Results were normalized to the wet biomass of the input tissue.

### Lung immune cell profiling

Lung tissue was minced using sterile scissors, incubated at 37°C for 30 minutes in RPMI-1640 (Sigma R0883) containing 10% fetal bovine serum (FBS; Gibco 10437-028), 0.1mg/mL DNase I (Sigma DN25), 0.8mg/mL dispase (Gibco 17105-041), and 1 mg/mL collagenase type 3 (Worthington Biochemical LS004183), and pushed through a 100μm filter to create a single-cell suspension. Cells were then washed twice in PBS, resuspended in 4 mL of RPMI-1640 with 10% FBS and 40% Percoll (Cytiva 17089101), and centrifuged at 2000 rpm for 30 minutes with no braking in order to pellet the immune cells. Cells were then resuspended in RPMI-1640 (Sigma R0883) with 10% FBS and GolgiPlug Protein Transport Inhibitor (1:1000, BD Biosciences 51-2301KZ) and stimulated for 2 hours with phorbol 12-myristate 13-acetate (PMA, 50ng/mL; Sigma P8139) and ionomycin (1μg/mL; Sigma I0634) at 37°C. After two hours, cells were washed twice in FACS buffer (PBS with 1% BSA), blocked for 15 minutes in PBS with 3% BSA, and incubated with Fixable Viability Dye eFluor™ 780 (1:3000; eBioscience 65-0865-14) and surface antibodies (see **Table S4**; anti-mouse IgG was used at 1:500, and all other antibodies were used at 1:200) at 4°C for 30 minutes in the dark. Cells were then washed twice in FACS buffer and incubated in Fix/Perm solution (eBioscience 00-5523-00) for 30 minutes at 4°C in the dark. Cells were then washed twice in permeabilization buffer (eBioscience 00-5523-00) and incubated with intracellular antibodies (see **Table S4**; all antibodies were used at 1:200) for 30 minutes at 4°C in the dark. Cells were then washed twice in permeabilization buffer, resuspended in FACS buffer, and analyzed on a Cytek Aurora flow cytometer.

### Detection of Spike-specific B and T cells

Spike-specific B and T cells were detected using biotinylated recombinant SARS-CoV-2 Spike receptor-binding domain (RBD; BioLegend 793904) labeled with streptavidin-Pacific Orange conjugate (SA-PO; Invitrogen S32365) prepared as previously described.^70^ Briefly, spike RBD was mixed with SA-PO at a 2:1 mass ratio/4:1 molar ratio (e.g. 25 ng RBD with 12.5 SA-PO) and incubated on ice for 1 hour in the dark. Excess biotin and SA-PO were removed using 7K MWCO Zeba Spin Desalting Columns (Thermo Fisher 89882). Cells were prepared and blocked as described above, then incubated for 30 minutes at 4°C in the dark with an antibody cocktail containing Fixable Viability Dye eFluor™ 780 (1:3000; eBioscience 65-0865-14); anti-CD45, CD3, CD4, CD8, B220, MHCII, and IgG antibodies (anti-IgG was used at 1:500 and all other antibodies at 1:200; see **Table S4**); and 25 ng SAPO-labeled Spike RBD per 50uL of antibody mix. Cells were then washed twice in FACS buffer and analyzed on a Cytek Aurora flow cytometer.

### IgG neutralization assay

Blood was collected from mice two-week post primary infection and purified using a Melon Gel IgG Spin Purification Kit (Thermo 45206) following manufacturer’s instructions. Purified IgG was diluted 1:20 and incubated with 1x10^5^ IU of rVSV-Spikeβ-GFP for 1 hour at 37°C before being added to Vero-TMPRSS2 cells at an MOI of 1. Cells were incubated at 37°C for 18 hours, washed once in PBS, and detached from the plate using TrypLE Express enzyme (Gibco 12604013). Cells were washed twice in PBS, incubated with in FACS buffer with Fixable Viability Dye eFluor™ 780 (1:3000; eBioscience 65-0865-14) for 30 minutes at 4°C in the dark, washed twice in FACS buffer, and analyzed using a Cytek Aurora flow cytometer.

### RNA-seq analysis of mouse lung tissue

Eight-week-old male GF mice were given control water or SCFA water for 2 weeks before lung tissue was harvested. Total RNA from whole lung tissue was isolated using the Qiagen RNeasy® Mini Kit (Qiagen 74104) according to the manufacturer’s instructions. Illumina stranded mRNA library prep, paired-end sequencing using the Illumina NovaSeq 6000 system, quality check, and sorting of reads with barcodes were performed by the Weill Cornell Medicine Genomics Resources Core Facility. Annotation, mapping, and counting of the resulted reads were analyzed by SALMON^71^ with default parameters and mouse cDNA sequences of GRCm38, which was obtained from Ensembl of European Molecular Biology Laboratory’s European Bioinformatics Institute, Cambridge, UK. To discover transcripts that exhibit differential abundance, we used LEfSe followed by multiple hypotheses correction using signal-to-noise ratio and false discovery rate analyses as described.^72^ Differences at LEfSe *p* < 0.05 and FDR <0.05 were considered significant, with negative linear discriminant analysis (LDA) scores indicating genes that were upregulated in GF mice and positive LDA scores indicating genes that were upregulated in SCFA-treated mice.

### Ex vivo human PBMC experiments

PBMCs were purified from blood using Ficoll-Paque (Cytiva 17144002) following manufacturer’s instructions. The purified cells were washed twice in PBS, then resuspended in RPMI-1640 with 10% FBS and penicillin–streptomycin (1:50; Sigma P4458). For SCFA treatments, a cocktail of sodium acetate, sodium butyrate, and sodium propionate was added to the culture media to a final concentration of 100μM sodium acetate, 50μM sodium butyrate, and 200μM sodium propionate. For HDAC inhibition, RGFP966 (Selleck Chemicals S7229) was added to the culture media to a final concentration of 5µM or 10µM. 5 × 10^5^ cells were used for each experimental condition. Cells were incubated at 37°C for 24 hours, after which they were washed twice in PBS and RNA was extracted and qPCR performed as described above.

### Megakaryocyte flow analysis

Bone marrow from control or SCFA-treated mice was flushed from femurs and tibias with sterile PBS, washed with FACS buffer, and pushed through a 100 μm filter to create a single-cell suspension. Cells were blocked in BD mouse Fc Block (1:100; BD Biosciences 553141) for 30 minutes at 4°C in the dark, washed in FACS buffer and incubated with anti-mouse c-Kit, CD41, and GPIbβ antibodies (all at 1:100; see **Table S4**) for 30 minutes at 4°C in the dark. Cells were then washed in FACS buffer, incubated with Fixable Viability Dye eFluor™ 780 (1:3000; eBioscience 65-0865-14), fixed with 1% paraformaldehyde (Thermo Scientific J19943-K2) and analyzed on a BD FACSymphony^TM^ A5 flow cytometer.

### Blood counts and D-Dimer analysis

At 3 d after infection, animals were euthanized via CO_2_ asphyxiation, and blood was immediately collected via cardiac puncture into syringes containing sodium citrate (Sigma 1613859) at a final concentration of 0.32% (w/v). Whole citrated blood was analyzed for complete blood count with automated differentials using a Beckman DxH 900 hematology analyzer (Beckman Coulter). Plasma was obtained by centrifuging citrated blood at 1000xg for 15 minutes at 4°C followed by 10,000xg centrifugation for 10 minutes at 4°C to remove all cells and debris. Plasma D-Dimer concentration was measured by a commercially available ELISA (LSBio LS-F6179) following the manufacturer’s instructions.

### Statistical analyses

All the statistical analyses were performed using Prism 9 (GraphPad Software, San Diego, CA).

Normality was determined using the Shapiro–Wilk normality test. Differences between two groups were evaluated using the unpaired *t*-test (for parametric data) or Mann–Whitney test (for nonparametric data), and comparisons of more than 3 groups were evaluated using ordinary one-way ANOVA followed by Tukey’s correction for multiple comparisons (for parametric data) or Kruskal–Wallis test followed by Dunn’s correction for multiple comparisons (for nonparametric data). Differences of *p*<0.05 were considered significant in all statistical analyses. Statistically significant differences are shown with asterisks as follows: *p<0.05, **p<0.01, ***p<0.001 and ****p<0.0001; comparisons which were nonsignificant are unmarked. Each figure shows data for individual animals or biological replicates.

## Supplementary Material

Supplemental Material

## Data Availability

Gene Expression Omnibus accession numbers for the publicly available RNA-seq datasets used in this study are as follows: Blanco-Melo et al. study^27^, GSE147507; Arunachalam et al. study^25^, GSE152418; Bernardes et al. study^26^, GSE161777. The GEO accession number for the 16S rRNA and RNA-seq data generated for this study is GSE189794.
