# Supplementary material for: Gut microbiota-derived metabolites confer protection against SARS-CoV-2 infection
Source: Gut Microbes. 2022 Aug 1;14(1):2105609. doi: 10.1080/19490976.2022.2105609 (PMC9348133; doi:10.1080/19490976.2022.2105609)
Supplement: Supplemental Material [file KGMI_A_2105609_SM2717.zip › Brown et al Gut Microbes supplemental 06242022.docx]

**Colon - *Clostridia* 16S rRNA abundance**

**Colon – total**

**16S rRNA abundance**

**Figure S1**

**A**

***Tmprss2***

**B**

**d**


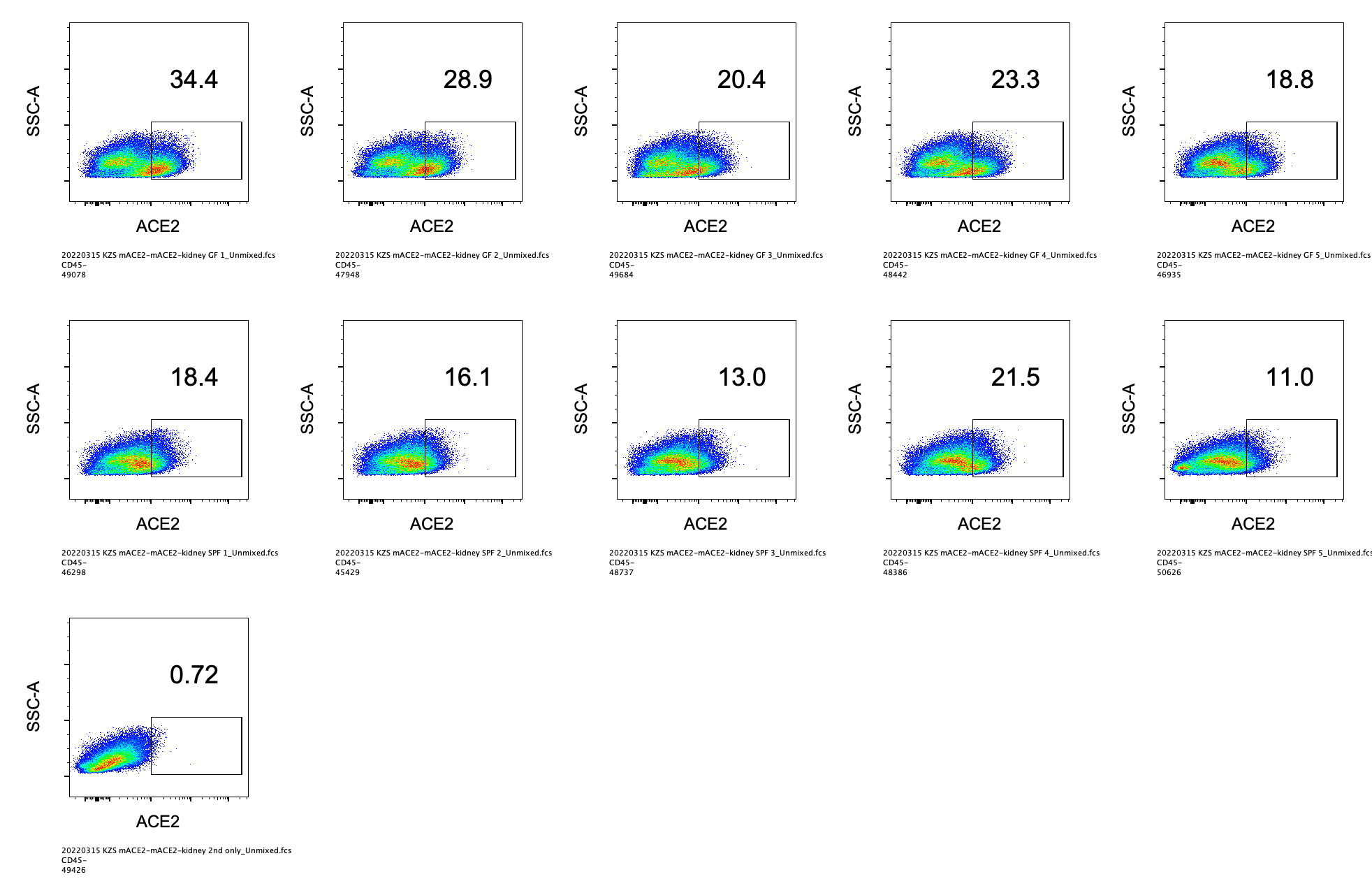

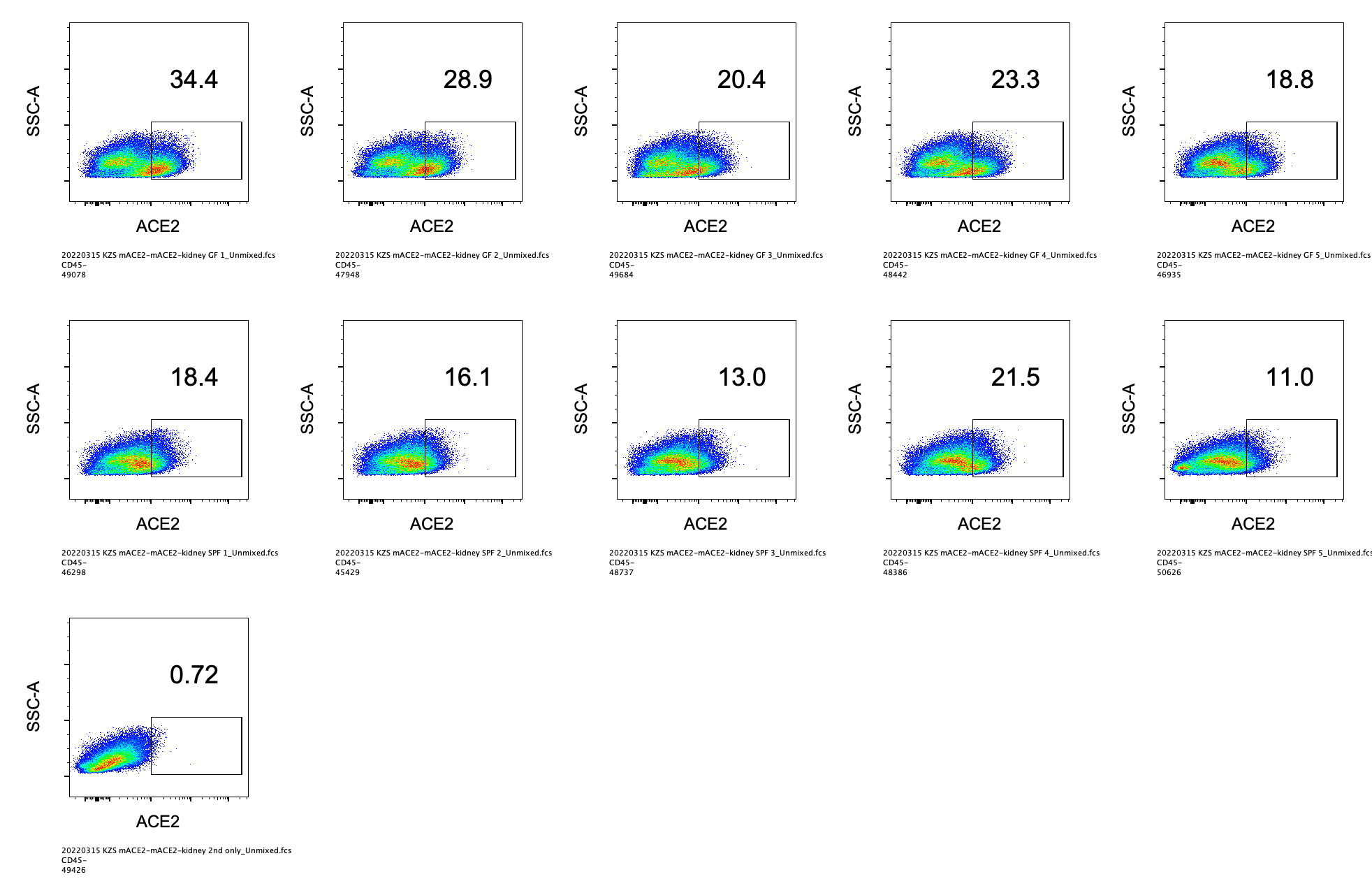

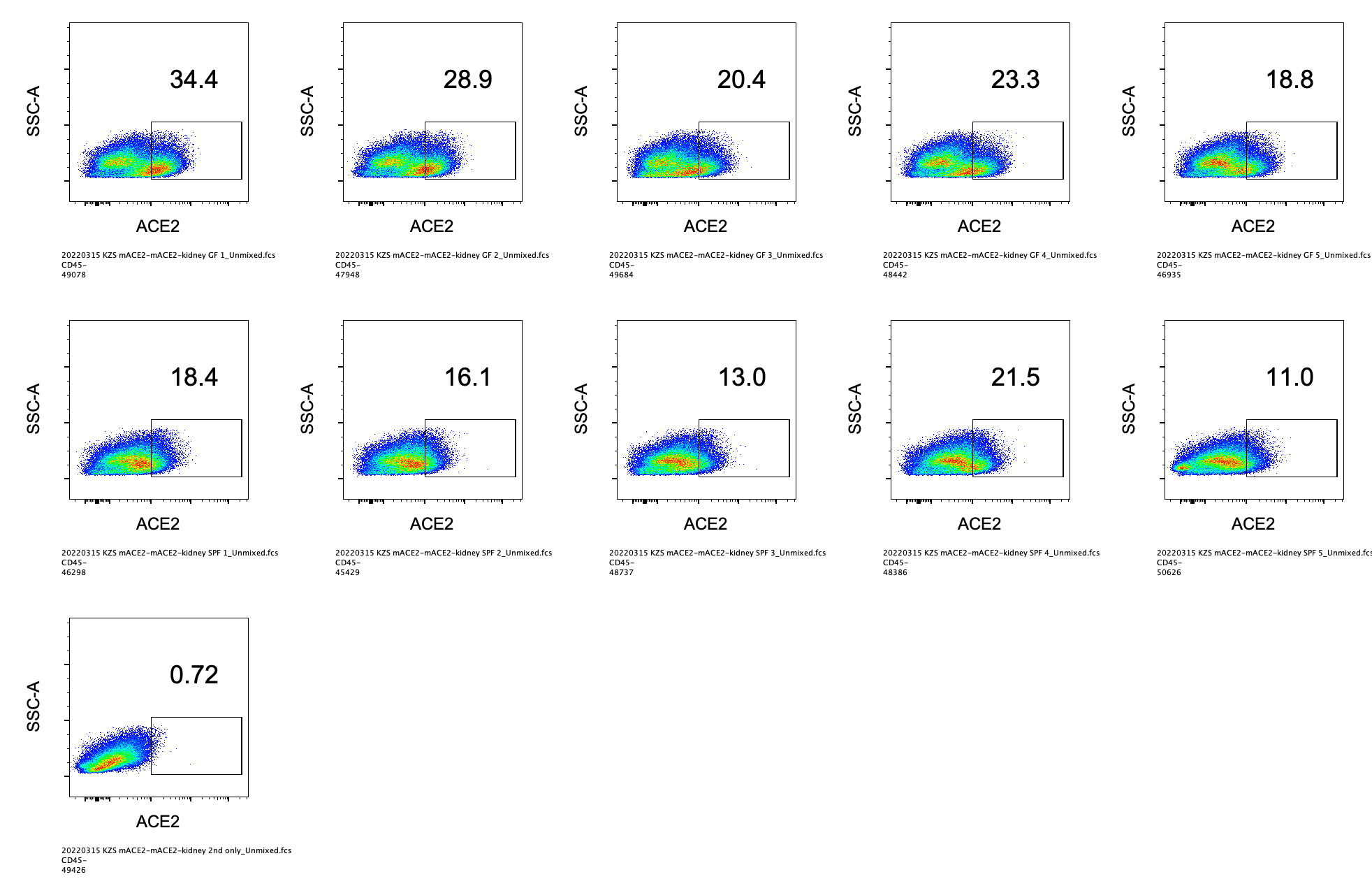


SSC-A

ACE2

**SPF**

**GF**

**2° Ab control**

Kidney CD45- cells:

**C**

***Tmprss2***

CONV

**D**

**E**

**Male**

**Female**

**Male**

**Female**

**F**

***Gpr43^-/-^***

**WT**

***Gpr41^-/-^***

***Gpr43^-/-^***

**WT**

***Gpr41^-/-^***

**Lung *Ace2* expression**

***Gpr41^-/-^***

***Gpr43^-/-^***

**WT**

**G**

**H**

Figure S1 (related to Figure 1). SCFAs modulate ACE2 expression in a sex-specific, GPR41- and GPR43-dependent manner.

(A) ACE2 expression in the kidney of 6-8 week old male SPF and GF adult mice was measured via flow cytometry. Quantifications and representative flow plots are shown. (B) *Tmprss2* gene expression in 6-8 week old male SPF and GF adult mice was measured by qPCR. (C-D) Male GF mice were colonized with either bulk fecal bacteria from SPF mice (CONV, conventionalized), or fecal bacteria enriched for *Clostridia*. After two weeks, (C) the total bacterial abundance and the relative abundance of *Clostridia* species in the colon was analyzed via 16S qPCR on colon luminal contents, and (D) *Tmprss2* expression in the indicated tissues was analyzed via qPCR. (E) SCFA concentrations were measured via LCMS analysis of fecal pellets from male SPF mice treated with gentamicin or vancomycin for two weeks. (F) Tmprss2 expression was measured via qPCR in the indicated tissues of male SPF, GF, or GF mice given SCFA drinking water for 2 weeks. (G) *Ace2* expression was measured via qRT-PCR in the indicated tissues of adult GF mice given control water or SCFA water for 2 weeks. (H) *Ace2* expression was measured via qPCR in the lungs and colons of wildtype, *Gpr41*^-/-^, *Gpr43*^-/-^, or *Gpr41*^-/-^*Gpr43*^-/-^ mice given control water or SCFA water for two weeks. SI = small intestine; FB = fecal bacteria. Error bars indicate mean±SEM. For (D-H), significance was determined using one-way ANOVA with Tukey’s test for multiple comparisons; for (A) and (B), significance was determined using unpaired t-test. For (C), significance was determined by unpaired t-test between the GF+FB and GF+Clostridia groups only. All data represent 2 independent experiments. **p*<0.05; ***p*<0.01; ****p*<0.001

**Figure S2**

**D**

**E**

**A**

**B**


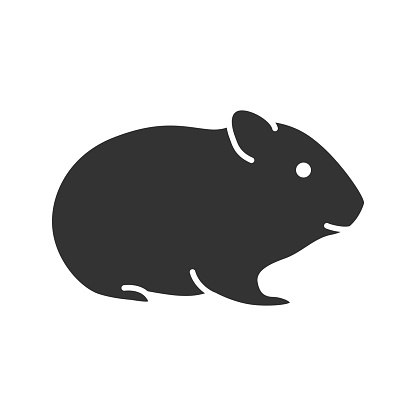


**Total plasma SCFAs –**

**SCFA-treated hamsters**

**Total plasma SCFAs –**

**pectin-treated mice**

**C**

**F**

***Ace2 – pectin-treated mice***

***Tmprss2 – pectin-treated mice***

**Lower lung - GF**

**Lower lung –**

**GF + SCFA**

**Upper lung - GF**

**Upper lung –**

**GF + SCFA**


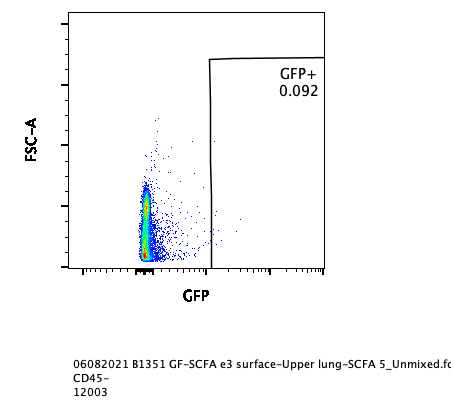

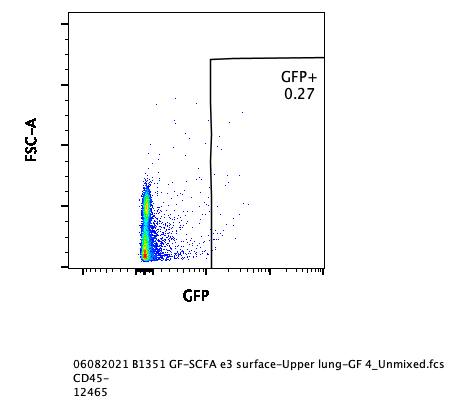

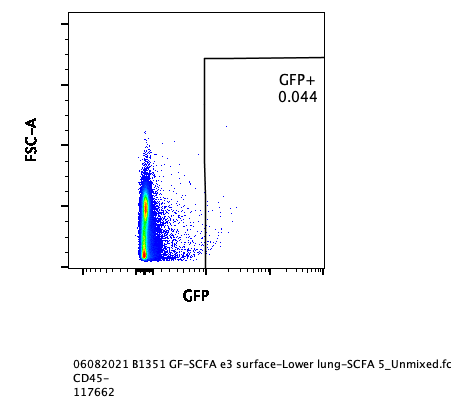

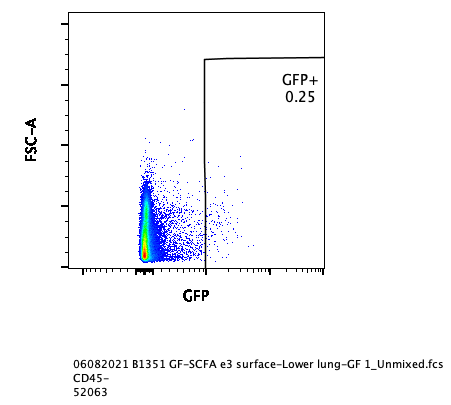

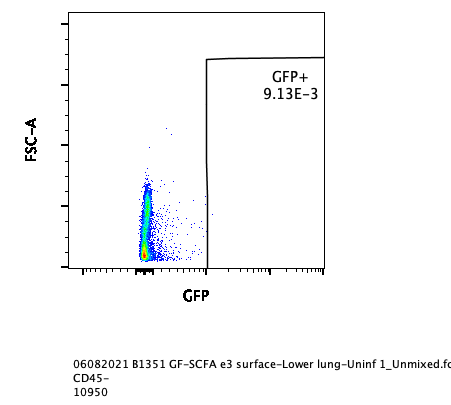


**Uninfected**

CD45- cells:


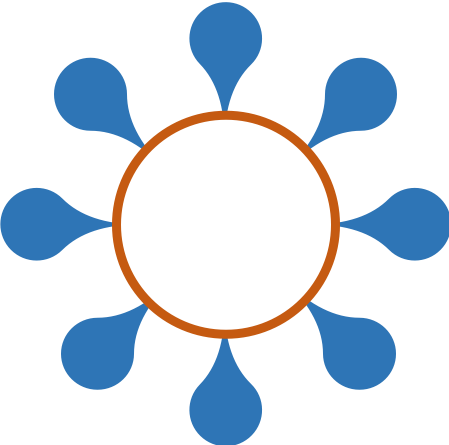


Replication-competent

rVSV-Spikeβ-GFP

Day: -14 0 3

Ctrl or SCFA water


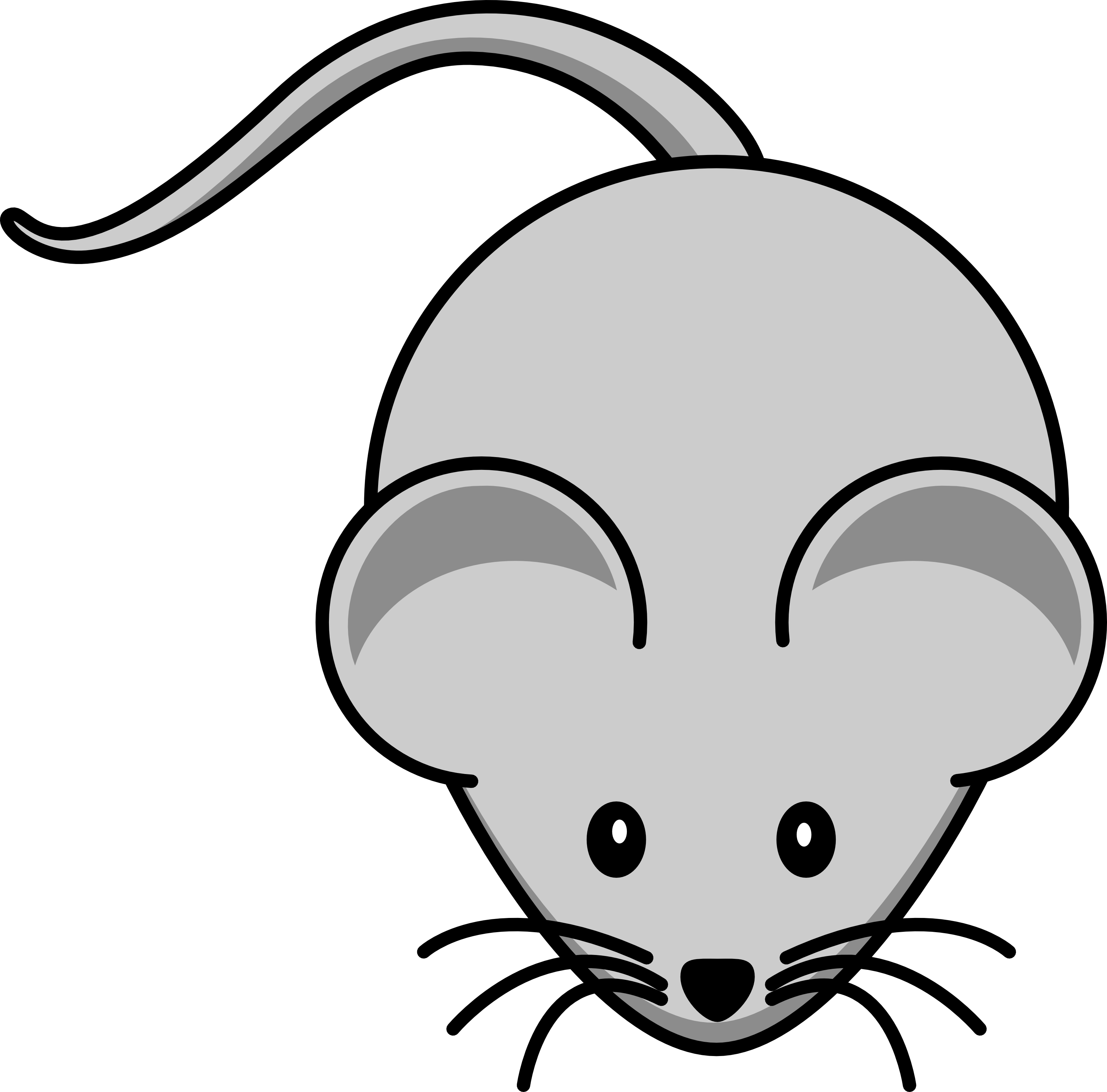


GF

Figure S2 (related to Figure 1 and Figure 2). Pectin diet suppresses *Ace2* and *Tmprss2* and promotes SCFA production, and SCFAs reduce viral burdens following intranasal infection with rVSV/Spikeβ-GFP.

(A) Human liver organoids were treated with SCFAs (low dose: 100µM acetate + 50µM butyrate + 200µM propionate; high dose: 1mM acetate + 0.5mM butyrate + 2mM propionate) for 24 hours before *Ace2* and *Tmprss2* expression was measured via qPCR. (B) *Ace2* and *Tmprss2* mRNA expression in SPF mice given a control diet or a diet with 5% or 30% pectin. (C) Plasma SCFA concentrations were measured via LCMS from male hamsters given a control diet or 30% pectin diet for two weeks. (D) Plasma SCFA concentrations were measured via LCMS from SPF mice given a control or 30% pectin diet for two weeks. (E) Plasma SCFA concentrations were measured via LCMS from hamsters given control or SCFA water for two weeks. (F) Germ-free mice were given regular water or SCFA water for two weeks before intranasal infection with replication-competent rVSV/Spikeβ-GFP. 72hrs following infection, viral burden in the upper and lower lungs was analyzed by measuring GFP-expressing cells via flow cytometry. Quantifications and representative flow cytometry plots are shown, gated on live CD45- cells. Error bars indicate mean±SEM. For (A-E), significance was determined using one-way ANOVA with Tukey’s test for multiple comparisons. For (F), significance was determined using unpaired t-test. **p*<0.05; ***p*<0.01; ****p*<0.001; *****p*<0.0001


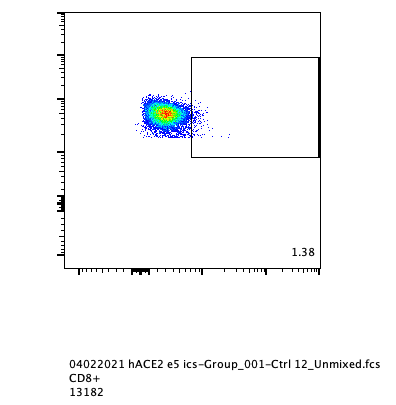

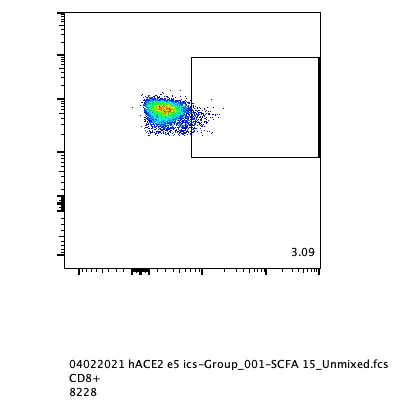

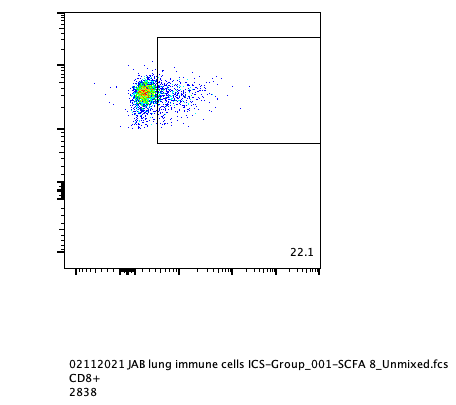

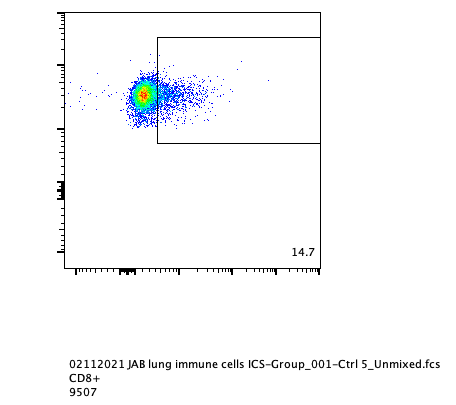

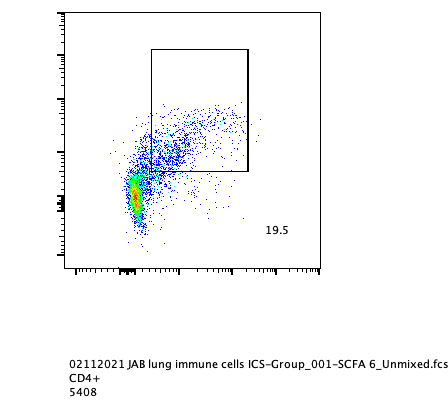

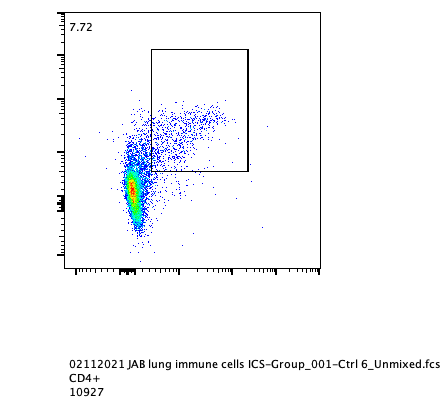


**Figure S3**

**A**

**B**

**Human *Ace2***

**Small intestine**

**Colon**


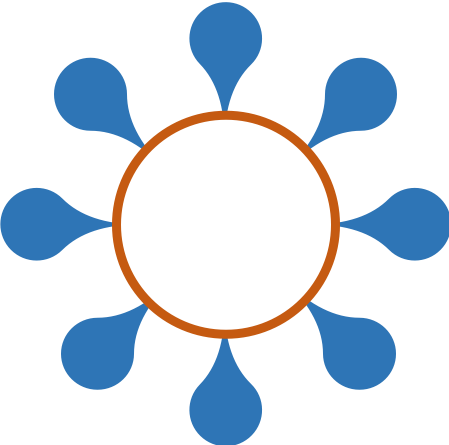


Replication-competent

rVSV-Spike-nLuc

Day: -14 0 3

Ctrl or SCFA water


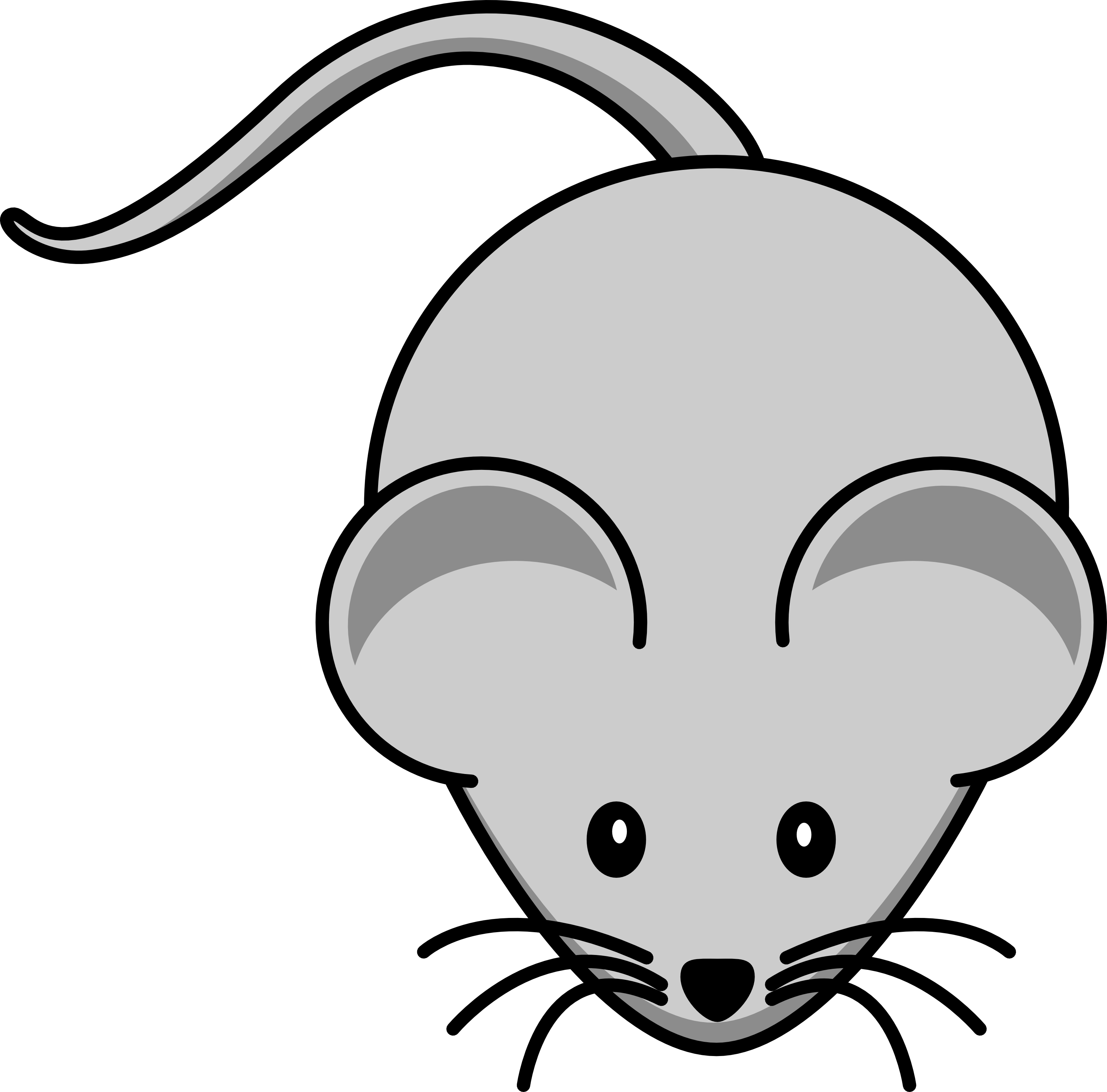


hACE2

**D**


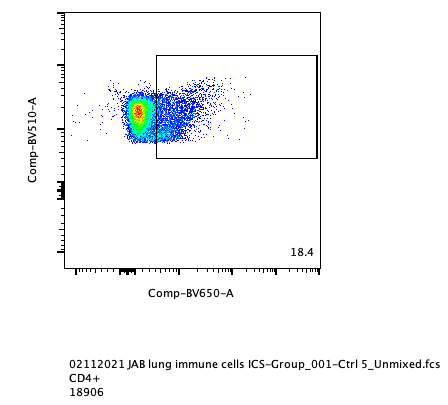

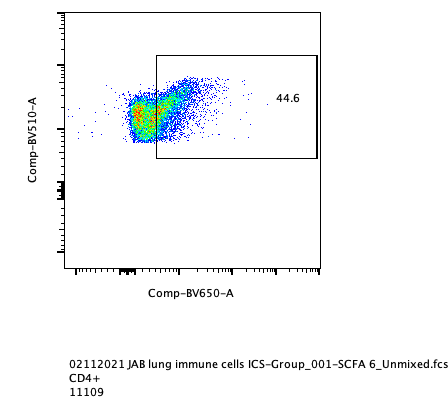


CD4

IFNγ

IFNγ+

18.4%

IFNγ+

44.6%

**Ctrl**

**SCFA**

CD4+ T cells

CD25

Foxp3

Tregs

7.72%

Tregs

19.5%

**Ctrl**

**SCFA**

CD8+ T cells

CD8

IFNγ

IFNγ+

14.7%

IFNγ+

22.1%

**Ctrl**

**SCFA**

CD8

GzmB

GzmB+

1.38%

GzmB+

3.09%

**Ctrl**

**SCFA**

**C**

Figure S3 (related to Figure 4). SCFAs alter the lung immune response following infection with rVSV/Spike-nLuc.

(A) hACE2 mice were treated with SCFA water for two weeks before human *Ace2* expression in the indicated tissues was measured via qPCR. (B-D) hACE2 mice were given control or SCFA water for two weeks before intranasal inoculation with rVSV/Spike-nLuc. 48h later, (B) viral burdens were determined by measuring luciferase activity, and (C-D) lung immune cells were measured via flow cytometry. Quantifications for (C) are shown in Figure 4B. RLUs = relative light units. Error bars indicate mean±SEM. For (A), significance was determined using one-way ANOVA with Tukey’s test for multiple comparisons; for all other panels, significance was determined using unpaired t-test. All data represent 3 independent experiments. **p*<0.05; ***p*<0.01

**A**

Day: -14 0 14 28 31


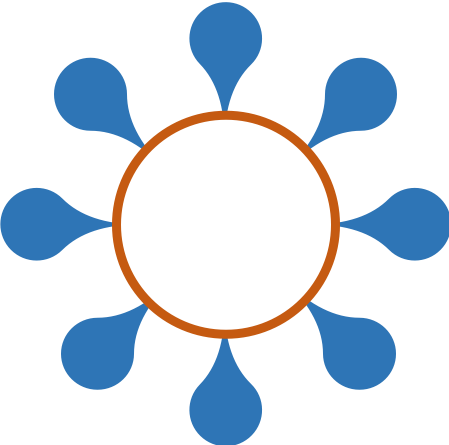


Ctrl or SCFA water


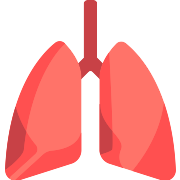


Replication-competent

rVSV-Spikeβ-GFP


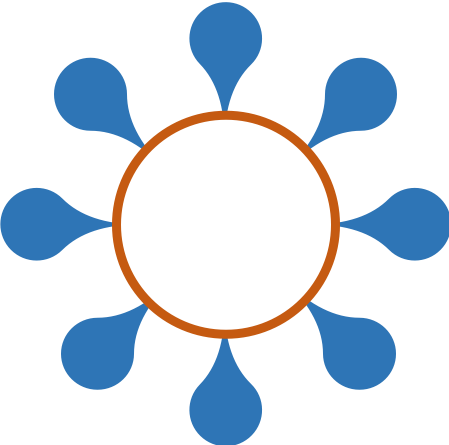


**B**

**A**

**B**

Day: -14 0 7 14


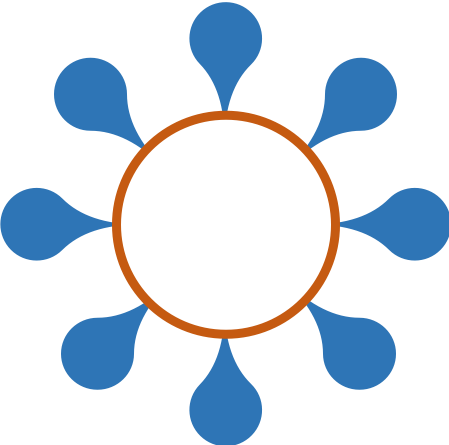


Ctrl or SCFA water

Heat-inactivated

rVSV-Spikeβ-GFP


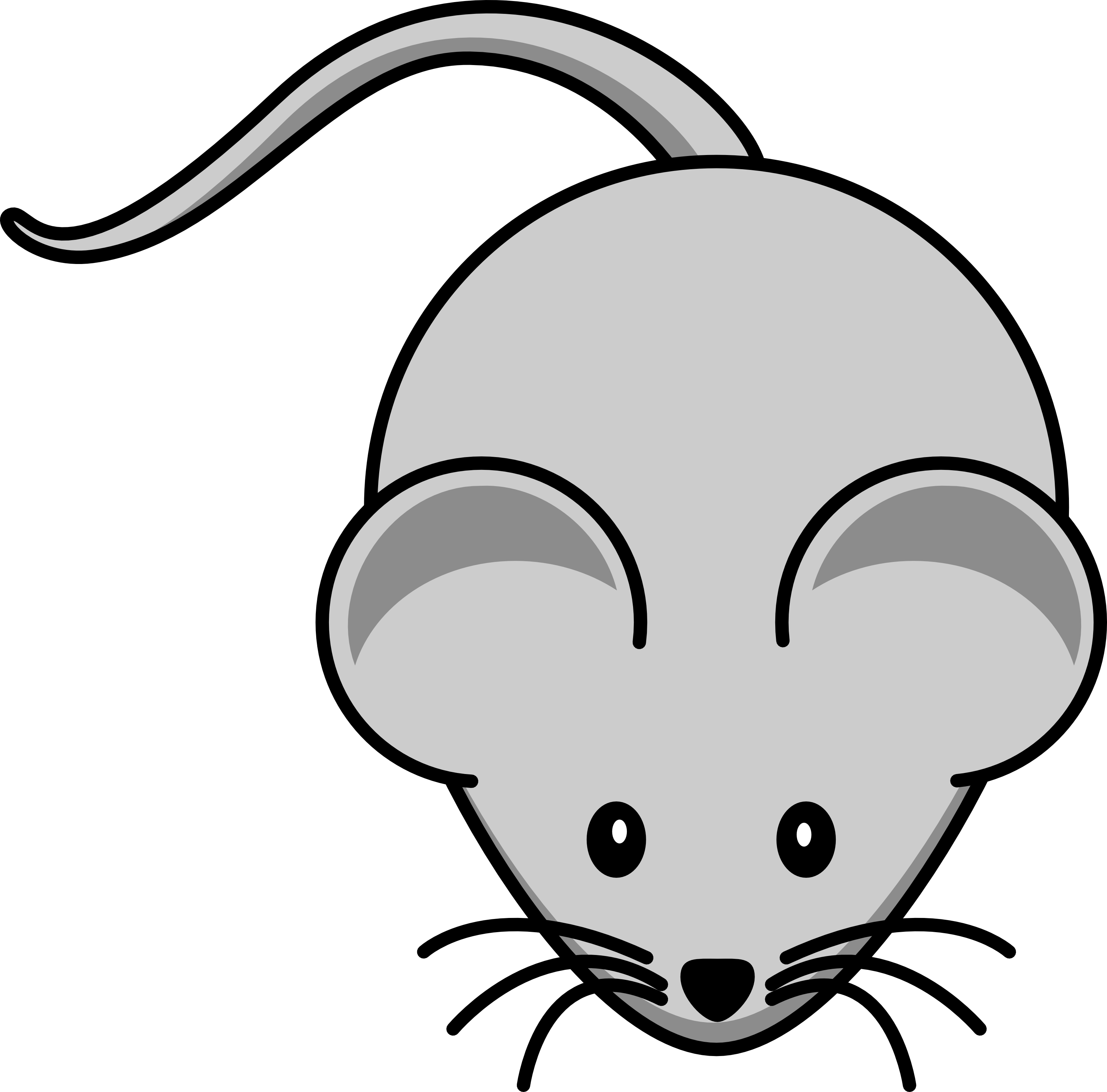


SPF WT


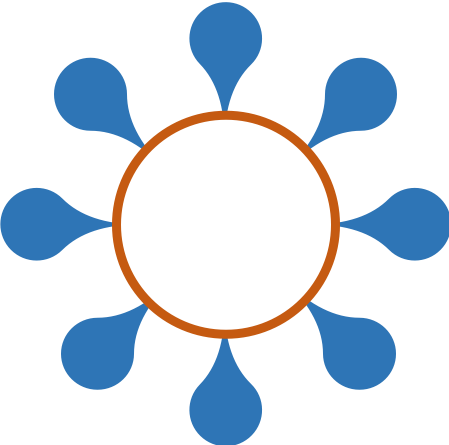


**C**

**D**

**E**

**Figure S4**


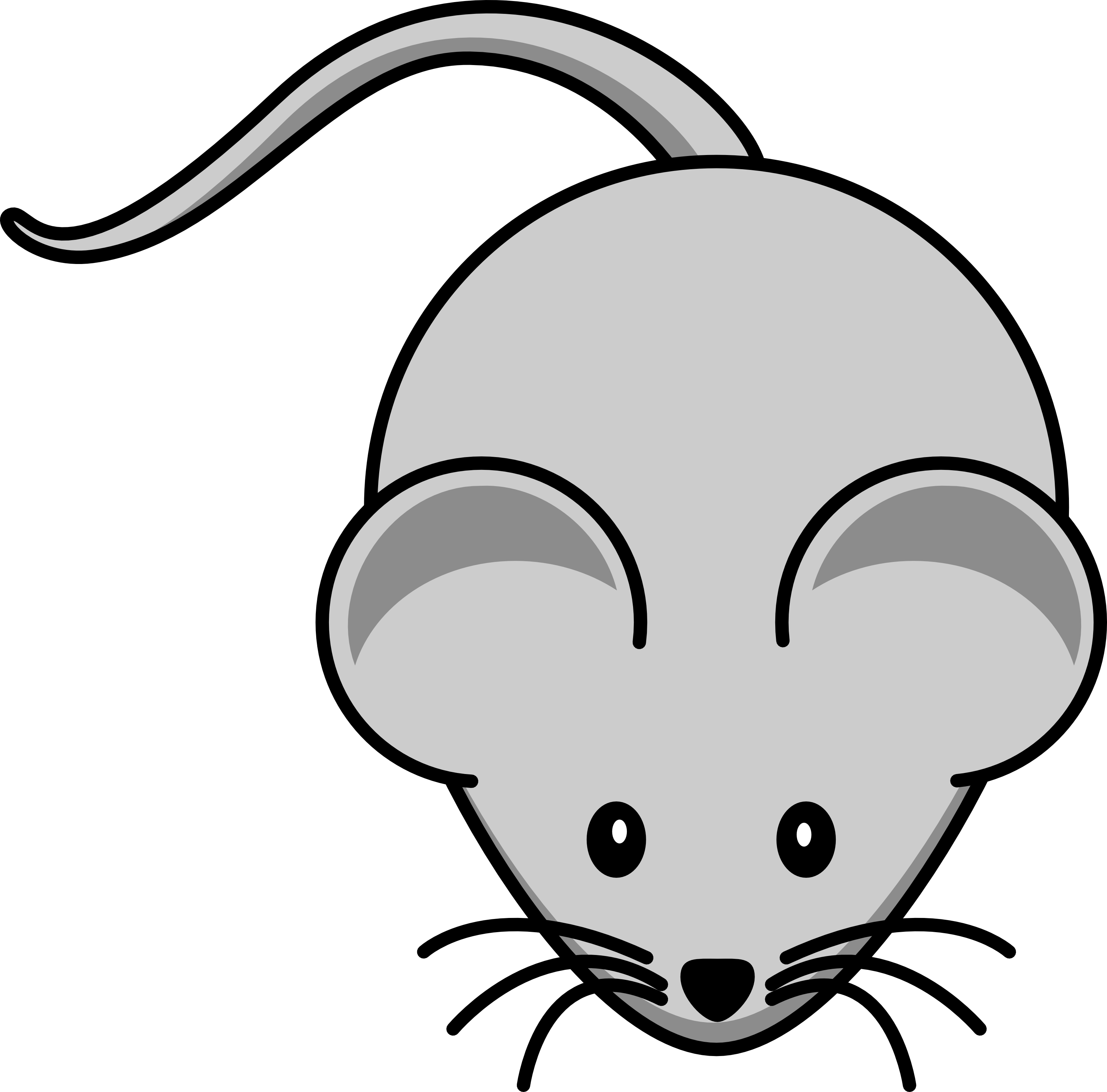


hACE2

**CD4+ T cells IFNγ+**

**Figure S4. SCFAs regulate the formation of immune memory in a sex-dependent manner.**

(**A-B**) hACE2 mice were given regular water or water with SCFAs for two weeks before intranasal infection with 6x10^4^ IU of replication-competent rVSV/Spikeβ-GFP. Blood was collected three days post infection for analysis of immune cells. Two weeks post infection, blood was collected and mice were reinfected with 5x10^5^ IU of the VSV-spike chimera. (**A**) Two weeks following the primary infection, IgG was purified from blood and preincubated with rVSV/Spikeβ-GFP for 1 hour before infecting Vero-TMPRSS2 cells. 24 hours later, the level of infection was determined by measuring GFP+ cells via flow cytometry. Data are shown as percentage of the no IgG condition. (**B**) Two weeks following the primary infection, blood was collected immediately prior to inoculation with the secondary infection and the immune cells were isolated and analyzed via flow cytometry. (**C-E**) (**C**) Male and female wild-type SPF mice were given control water or SCFA water for two weeks before intranasal inoculation with 10^6^ IU of heat inactivated rVSV/Spikeβ-GFP . A second inoculation was given after one week. After a further week, (**D**) blood immune cells were analyzed via flow cytometry, and (**E**) plasma was preincubated with rVSV/Spikeβ-GFP before infecting Vero-TMPRSS2 cells. 24 hours later, GFP+ cells were measured via flow cytometry. Data are shown as percentage of the no plasma condition. Data are shown combined and separated by sex. Error bars indicate mean±SEM. For (**A**) and (**E**), significance was determined using one-way ANOVA with Tukey’s test for multiple comparisons; for all other panels, significance was determined using unpaired t-test. Data represent 2 independent experiments. **p*<0.05; ***p*<0.01; *****p*<0.0001

**Figure S5**

**A**

**B**

**C**

**D**

**F**

**E**

***Gpr43^-/-^***

**WT**

***Gpr41^-/-^***

**Male**

**Female**

**Female**

**Male**

***Gpr41^-/-^Gpr43^-/-^***

**Megakaryocytes**

***Gpr41^-/-^Gpr43^-/-^***

**Mean platelet volume**

***Gpr41^-/-^Gpr43^-/-^***

**Plasma D-dimer**

**Figure S5 (related to Figure 5). SCFAs dampen the coagulation response.**

(**A**) A complete blood count was performed on blood from male GF mice given control water or SCFA water for two weeks. (**B**) *Sh2b3* mRNA expression in the lungs of male wildtype, *Gpr41*^-/-^, or *Gpr43*^-/-^ mice given control water or SCFA water for two weeks. (**C**) *Gpr41*^-/-^*Gpr43*^-/-^ mice were given control water or SCFA water for two weeks before a complete blood count was performed, plasma D-dimer was measured by ELISA, and bone marrow megakaryocytes were measured via flow cytometry. (**D**) *Sh2b3* mRNA expression in PBMCs from healthy human donors treated with SCFAs. (**E**) *Sh2b3* mRNA expression in the lungs of male and female GF mice given control or SCFA water for two weeks. (**F**) Male GF mice were given control or SCFA water for two weeks before *Sh2b3* and *TPO* expression was measured in the liver. Error bars indicate mean±SEM. For (**B**), (**D**), and (**E**), significance was determined using one-way ANOVA with Tukey’s test for multiple comparisons; for all other panels, significance was determined using unpaired t-test. (**A**) and (**C**) represent 2 independent experiments; (**D-F**) represent 3 independent experiments. **p*<0.05; ***p*<0.01

**Table S1 (related to Figure 5). Significantly differentially regulated genes in GF or SCFA-treated mice.** Negative linear discriminant analysis (LDA) scores indicate genes that are upregulated in GF mice and positive LDA scores indicate genes that are upregulated in SCFA-treated mice.

| **Gene** | **Linear discriminant analysis score** | **-Log_10_(P value)** |
| --- | --- | --- |
| Pim3 | 2.7 | 4.8563 |
| Tmpo | 1.54 | 4.7971 |
| Dicer1 | 1.76 | 4.7812 |
| **Sh2b3** | **0.89** | **4.4277** |
| Srsf4 | 1.33 | 4.0599 |
| Hnrnpa2b1 | 0.84 | 3.9595 |
| Stx1a | 0.61 | 3.7655 |
| Crtc2 | 1.02 | 3.7647 |
| Zfp638 | -2.15 | 3.759 |
| Tmem8 | 0.72 | 3.6875 |
| Rexo1 | 0.76 | 3.6558 |
| Kank3 | 1.87 | 3.5445 |
| Par1 | 0.17 | 3.3949 |
| Sh3glb2 | -36.7 | 3.3022 |
| Pprc1 | 1.95 | 3.2941 |
| Fbx116 | 0.02 | 3.2494 |
| Arhgap12 | 0.79 | 3.2397 |
| Myo1b | 0.65 | 3.2227 |
| Traf7 | 1.97 | 3.2192 |
| AI467606 | 0.76 | 3.2177 |
| Zbtb42 | 0.8 | 3.1833 |
| Fbrs | 1.27 | 3.1783 |
| Slc18b1 | -0.3 | 3.1447 |
| Tlcd1 | 0.77 | 3.1172 |
| Banp | 3.75 | 3.0995 |
| Yif1b | 0.71 | 3.0823 |
| Map3k15 | 1.7 | 3.0822 |
| Mcl1 | 0.74 | 3.0781 |
| Nkx2-9 | -23.4 | 3.0745 |
| Fbxo2 | 1.43 | 3.0585 |
| Rin3 | 0.54 | 3.0366 |
| Pigm | 0.61 | 3.0347 |

**Table S2**. **Pectin diet formulations.**

| **Component** | **Control diet (AIN93G)** | | **5% pectin diet** | | **30% pectin diet** | |
| --- | --- | --- | --- | --- | --- | --- |
|  | **gm** | **kcal** | **gm** | **kcal** | **gm** | **kcal** |
| Casein | 200 | 800 | 200 | 800 | 200 | 800 |
| L-Cysteine | 3 | 12 | 3 | 12 | 3 | 12 |
| Cornstarch | 397.486 | 1590 | 397.486 | 1590 | 397.486 | 1590 |
| Maltodextrin 10 | 132 | 528 | 132 | 528 | 132 | 528 |
| Sucrose | 100 | 400 | 100 | 400 | 100 | 400 |
| **Cellulose, BW200** | **50** | **0** | **0** | **0** | **0** | **0** |
| **Pectin, 1400 (80% Fiber)** | **0** | **0** | **50** | **0** | **407.2** | **0** |
| Soybean Oil | 70 | 630 | 70 | 630 | 70 | 630 |
| t-Butylhydroquinone | 0.014 | 0 | 0.014 | 0 | 0.014 | 0 |
| Mineral mix S10022G | 35 | 0 | 35 | 0 | 35 | 0 |
| Vitamin mix V10037 | 10 | 40 | 10 | 40 | 10 | 40 |
| Choline Bitartrate | 2.5 | 0 | 2.5 | 0 | 2.5 | 0 |
| FD&C Blue Dye #1 | 0 | 0 | 0.05 | 0 | 0 | 0 |
| FD&C Red Dye #40 | 0 | 0 | 0 | 0 | 0.05 | 0 |
| **Total** | **1000** | **4000** | **1000.05** | **4000** | **1357.25** | **4000** |

**Table S3. List of primers.**

| **Gene** | **Forward primer (5’-3’)** | **Reverse primer (5’-3’)** | **Reference** |
| --- | --- | --- | --- |
| Mouse ACE2 | TCCAGACTCCGATCATCAAGC | TGCTCATGGTGTTCAGAATTGT | ^1^ |
| Mouse β-actin | AAGGCCAACCGTGAAAAGAT | GTGGTACGACCAGAGGCATAC | ^2^ |
| Mouse Sh2b3/Lnk | ACAGCCAACAGAGCTGAGGT | CGTTCTCCTATCCCACTGGA | ^3^ |
| Mouse TMPRSS2 | CAGTCTGAGCACATCTGTCCT | CTCGGAGCATACTGAGGCA | ^4^ |
| Mouse TPO | CTCTGTCCAGCCCCGTAGC | CCCCAAGAGGAGGCGAAC | ^5^ |
| Human ACE2 | CGAAGCCGAAGACCTGTTCTA | GGGCAAGTGTGGACTGTTCC | ^6^ |
| Human β-actin | GCAAGCAGGACTATGACGAG | CAAATAAAGCCATGCCAATC | ^7^ |
| Human Mpl | CCAGCCAGGGGAACTTC | GCTTTGGTCCATCTTGCC | ^8^ |
| Human Sh2b3 | GCTCAACACCAAACTGGACAGTAGA | CCGGGAGCTGTCAAGCTGTA | ^9^ |
| Human TMPRSS2 | GTCCCCACTGTCTACGAGGT | CAGACGACGGGGTTGGAAG | ^10^ |
| 16S rRNA | CCTACGGGTGGCTGCAG | GACTACTAGGGTATCTAATCC | ^11^ |
| Clostridia 16S rRNA | ACTCCTACGGGAGGCAGC | GCTTCTTTAGTCAGGTACCGTCAT | ^12^ |
| SARS-CoV-2 IP2 primers | ATGAGCTTAGTCCTGTTG | CTCCCTTTGTTGTGTTGT | ^13^ |
| SARS-CoV-2 RdRP probe | AGATGTCTTGTGCTGCCGGTA [5']Hex [3']BHQ-1 |  | ^13^ |

**Table S4 (related to Methods). Antibodies.**

| **Antibodies** | **Clone** | **Source** | **Identifier** |
| --- | --- | --- | --- |
| Goat anti-mouse ACE-2 antibody | Polyclonal | R&D Systems | AF3437 |
| Rat anti-mouse ACE-2 antibody | 460502 | R&D Systems | MAB3437 |
| THE™ beta actin antibody [HRP] | 2D1D10 | GenScript | A00730 |
| Goat anti-rat IgG (H+L) Secondary Antibody, HRP | Polyclonal | Invitrogen | 31470 |
| Donkey anti-goat IgG H&L (Alexa Fluor® 488) | Polyclonal | Abcam | ab150129 |
| Alexa Fluor-700 anti-mouse CD45 | 30-F11 | BioLegend | 103128 |
| Brilliant Violet 711 anti-mouse CD3e | 145-2C11 | BioLegend | 100349 |
| Brilliant Violet 510 anti-mouse CD4 | RM4-4 | BioLegend | 116025 |
| PerCP-eFluor 710 anti-mouse CD8a | 53-6.7 | Invitrogen | 46-0081-80 |
| PE anti-mouse CD25 | PC61 | BioLegend | 102007 |
| Brilliant Violet 650 anti-mouse IFN-γ | XMG1.2 | BioLegend | 505832 |
| FITC anti-human/mouse Granzyme B | QA18A28 | BioLegend | 396404 |
| APC anti-mouse perforin | S16009B | BioLegend | 154404 |
| PE-Cyanine5.5 anti-mouse/rat Foxp3 | FJK-16s | Invitrogen | 35-5773-82 |
| PE/Cyanine7 anti-mouse TNF-⍺ | MP6-XT22 | BioLegend | 506323 |
| PE-Cyanine7 anti-mouse Ly-6G (Gr1) | RB6-8C5 | eBioscience | 25-5931-82 |
| PE anti-mouse/human CD11b | M1/70 | BioLegend | 101208 |
| FITC anti-mouse CD11c | N418 | BioLegend | 117305 |
| Pacific Blue anti-mouse/human CD45R/B220 | RA3-6B2 | BioLegend | 103227 |
| BUV395 anti-mouse CD86 | GL1 | BD Biosciences | 564199 |
| Brilliant Violet 421 anti-mouse IL-17A | TC11-18H10.1 | BioLegend | 506926 |
| PE-Cyanine7 anti-mouse Ly-6G (Gr1) | 1A8 | BioLegend | 127618 |
| APC-eFluor 780 anti-mouse IFNγ | XMG1.2 | Invitrogen | 47-7311-80 |
| PerCP-Cyanine5.5 anti-mouse CD11b | M1/70 | BD Biosciences | 550993 |
| APC anti-mouse IgG (H+L) | Polyclonal | Invitrogen | 17-4010-82 |
| APC anti-mouse MHCII I-A/I-E | M5/114.15.2 | BioLegend | 107614 |
| Pacific Blue anti-mouse CD11b | M1/70 | BioLegend | 101224 |
| PE-Cyanine5 anti-mouse IgM | II/41 | eBioscience | 15-5790-81 |
| Brilliant Violet 650 anti-mouse MHCII I-A/I-E | M5/114.15.2 | BioLegend | 107641 |
| APC-eFluor 780 anti-mouse CD45R/B220 | RA3-6B2 | Invitrogen | 47-0452-82 |
| PE/Cyanine5 anti-mouse CD11c | N418 | BioLegend | 117316 |
| PerCP/Cyanine5.5 anti-mouse MHCII I-A/I-E | M5/114.15.2 | BioLegend | 107625 |
| APC anti-mouse F4/80 | BM8 | Invitrogen | 17-4801-82 |
| PE anti-mouse CD117 (c-Kit) | 2B8 | BioLegend | 105808 |
| Alexa Fluor 488 anti-mouse CD41 | MWReg30 | BioLegend | 133908 |
| DyLight anti-mouse GPIbβ | Anti-GPIbβ derivative | Emfret | X649 |

**References**

1. Burgueño JF, Reich A, Hazime H, Quintero MA, Fernandez I, Fritsch J, Santander AM, Brito N, Damas OM, Deshpande A, et al. 2020. Expression of SARS-CoV-2 Entry Molecules ACE2 and TMPRSS2 in the Gut of Patients With IBD. Inflamm Bowel Dis [Internet]. [accessed 2021 Jul 21] 26(6):797. doi:10.1093/IBD/IZAA085

2. Hohenstein P, Slight J, Ozdemir DD, Burn SF, Berry R, Hastie ND. 2008. High-efficiency Rosa26 knock-in vector construction for Cre-regulated overexpression and RNAi. Pathogenetics [Internet]. [accessed 2021 Jul 21] 1(1):3. doi:10.1186/1755-8417-1-3

3. Lee JH, Lee SH, Lee HS, Ji ST, Jung SY, Kim JH, Bae SS, Kwon S-M. 2016. Lnk is an important modulator of insulin-like growth factor-1/Akt/peroxisome proliferator-activated receptor-gamma axis during adipogenesis of mesenchymal stem cells. Korean J Physiol Pharmacol [Internet]. [accessed 2021 Aug 25] 20(5):459. doi:10.4196/KJPP.2016.20.5.459

4. Bilinska K, Jakubowska P, Bartheld CS Von, Butowt R. 2020. Expression of the SARS-CoV-2 Entry Proteins, ACE2 and TMPRSS2, in Cells of the Olfactory Epithelium: Identification of Cell Types and Trends with Age. ACS Chem Neurosci [Internet]. [accessed 2021 Jul 21] 11(11):1555–1562. doi:10.1021/ACSCHEMNEURO.0C00210

5. Burmester H, Wolber E-M, Freitag P, Fandrey J, Jelkmann DW. 2005. Thrombopoietin Production in Wild-Type and Interleukin-6 Knockout Mice with Acute Inflammation. J Interf Cytokine Res [Internet]. [accessed 2021 Oct 1] 25(7):407–413. doi:10.1089/JIR.2005.25.407

6. Lamers MM, Beumer J, Vaart J Van Der, Knoops K, Puschhof J, Breugem TI, Ravelli RBG, Schayck JP Van, Mykytyn AZ, Duimel HQ, et al. 2020. SARS-CoV-2 productively infects human gut enterocytes. Science (80- ) [Internet]. [accessed 2022 Apr 15] 369(6499):50–54. doi:10.1126/science.abc1669

7. Zeng Z, Li M, Chen J, Li Q, Ning Q, Zhao J, Xu Y, Xie J, Yu J. 2018. Reduced MBD2 expression enhances airway inflammation in bronchial epithelium in COPD. Int J Chron Obstruct Pulmon Dis [Internet]. [accessed 2021 Aug 25] 13:703. doi:10.2147/COPD.S148595

8. Pulikkan JA, Madera D, Xue L, Bradley P, Landrette SF, Kuo Y-H, Abbas S, Zhu LJ, Valk P, Castilla LH. 2012. Thrombopoietin/MPL participates in initiating and maintaining RUNX1-ETO acute myeloid leukemia via PI3K/AKT signaling. Blood [Internet]. [accessed 2021 Oct 1] 120(4):868. doi:10.1182/BLOOD-2012-03-414649

9. Hao M, Yuan F, Jin C, Zhou Z, Cao Q, Xu L, Wang G, Huang H, Yang D, Xie M, Zhao X. 2016. Overexpression of Lnk in the Ovaries Is Involved in Insulin Resistance in Women With Polycystic Ovary Syndrome. Endocrinology [Internet]. [accessed 2021 Aug 25] 157(10):3709. doi:10.1210/EN.2016-1234

10. Vidal SJ, Rodriguez-Bravo V, Quinn SA, Rodriguez-Barrueco R, Lujambio A, Williams E, Sun X, DelaIglesia-Vicente J, Lee A, Readhead B, et al. 2015. A targetable GATA2-IGF2 axis confers aggressiveness in lethal prostate cancer. Cancer Cell [Internet]. [accessed 2022 Apr 15] 27(2):223–239. doi:10.1016/J.CCELL.2014.11.013

11. Sambo F, Finotello F, Lavezzo E, Baruzzo G, Masi G, Peta E, Falda M, Toppo S, Barzon L, Di Camillo B. 2018. Optimizing PCR primers targeting the bacterial 16S ribosomal RNA gene. BMC Bioinformatics [Internet]. [accessed 2021 Aug 26] 19(1):1–10. doi:10.1186/S12859-018-2360-6

12. Rivera-Chávez F, Zhang LF, Faber F, Lopez CA, Byndloss MX, Olsan EE, Xu G, Velazquez EM, Lebrilla CB, Winter SE, Bäumler AJ. 2016. Depletion of butyrate-producing Clostridia from the gut microbiota drives an aerobic luminal expansion of Salmonella. Cell Host Microbe [Internet]. [accessed 2021 Aug 26] 19(4):443. doi:10.1016/J.CHOM.2016.03.004

13. Etievant S, Bal A, Escuret V, Brengel-Pesce K, Bouscambert M, Cheynet V, Generenaz L, Oriol G, Destras G, Billaud G, et al. 2020. Performance Assessment of SARS-CoV-2 PCR Assays Developed by WHO Referral Laboratories. J Clin Med [Internet]. [accessed 2022 Jun 1] 9(6):1–10. doi:10.3390/JCM9061871
