# Supplementary figures and images for: Gut microbiota-derived metabolites confer protection against SARS-CoV-2 infection
Source: Gut Microbes. 2022 Aug 1;14(1):2105609. doi: 10.1080/19490976.2022.2105609 (PMC9348133; doi:10.1080/19490976.2022.2105609)

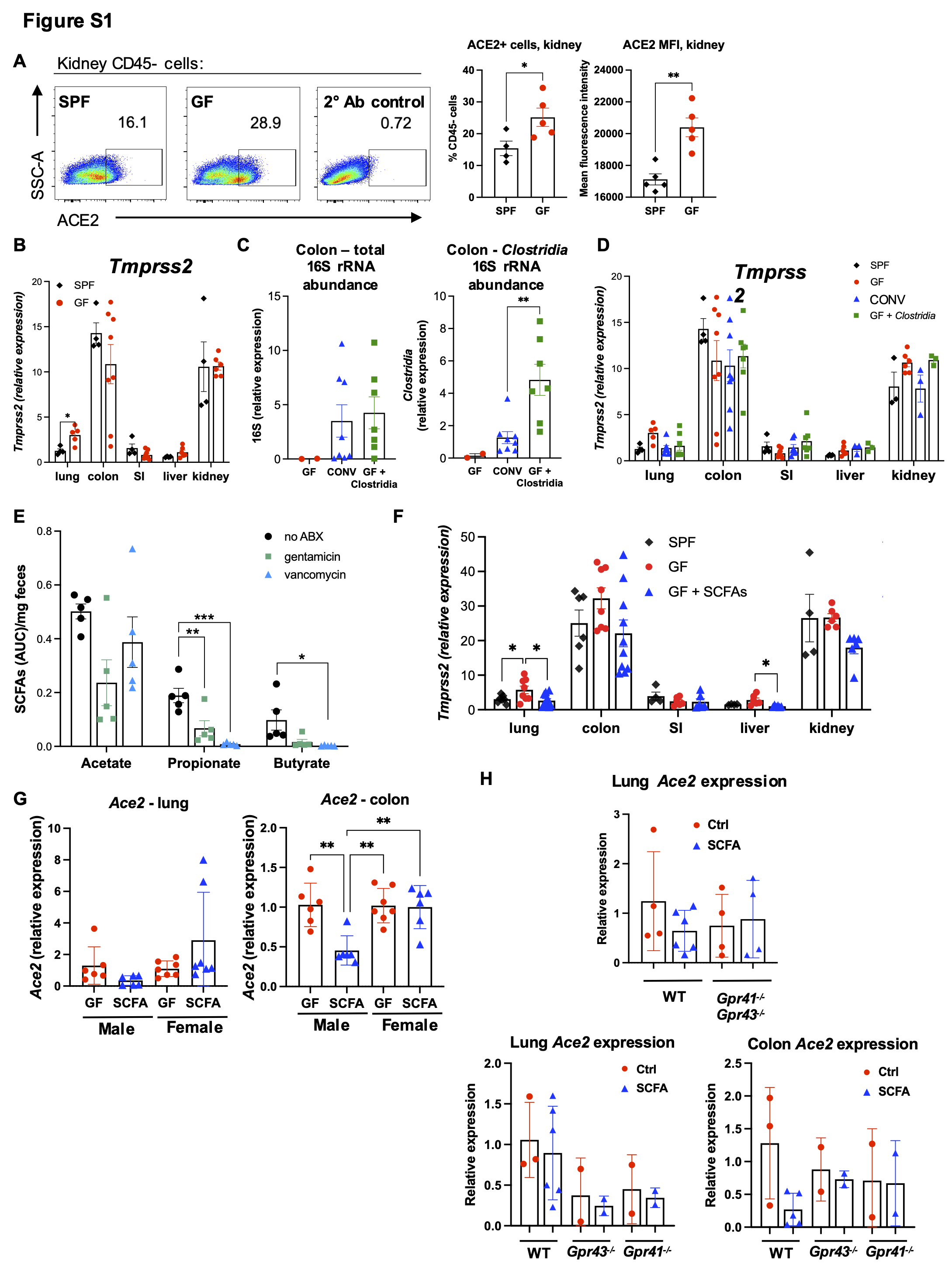

Supplement: Supplemental Material [file KGMI_A_2105609_SM2717.zip › Figure S1.tiff]

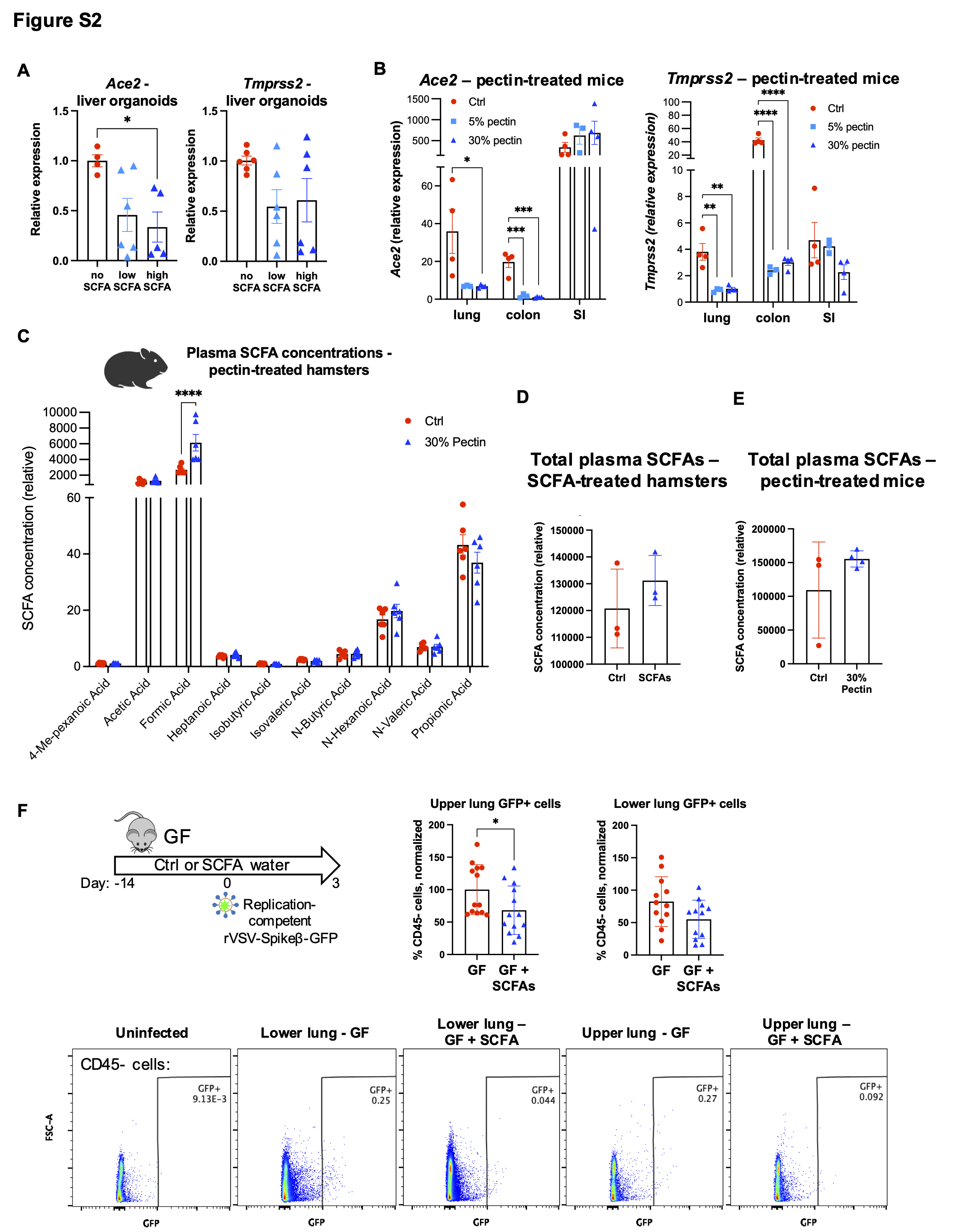

Supplement: Supplemental Material [file KGMI_A_2105609_SM2717.zip › Figure S2.tiff]

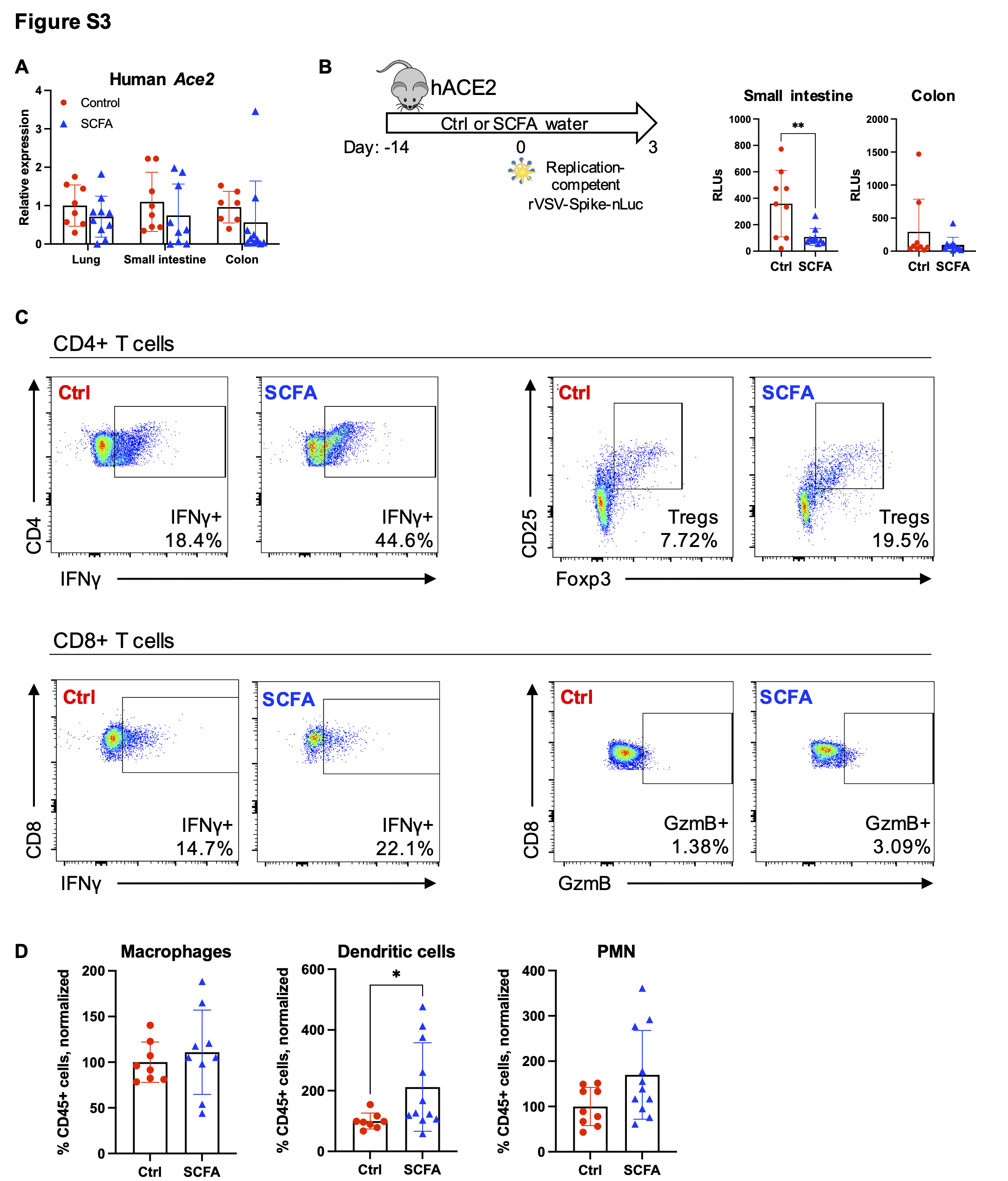

Supplement: Supplemental Material [file KGMI_A_2105609_SM2717.zip › Figure S3.tiff]

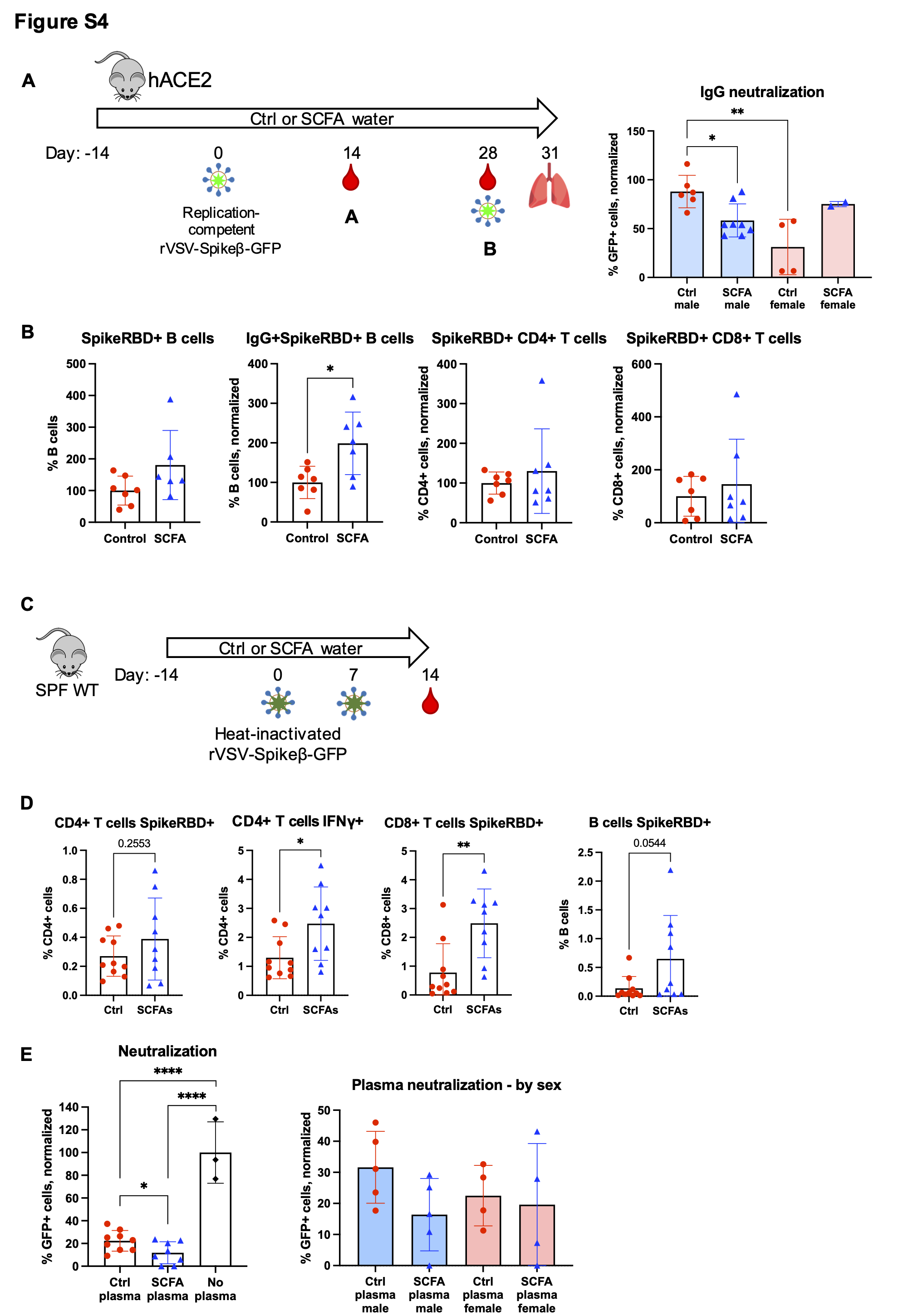

Supplement: Supplemental Material [file KGMI_A_2105609_SM2717.zip › Figure S4.tiff]

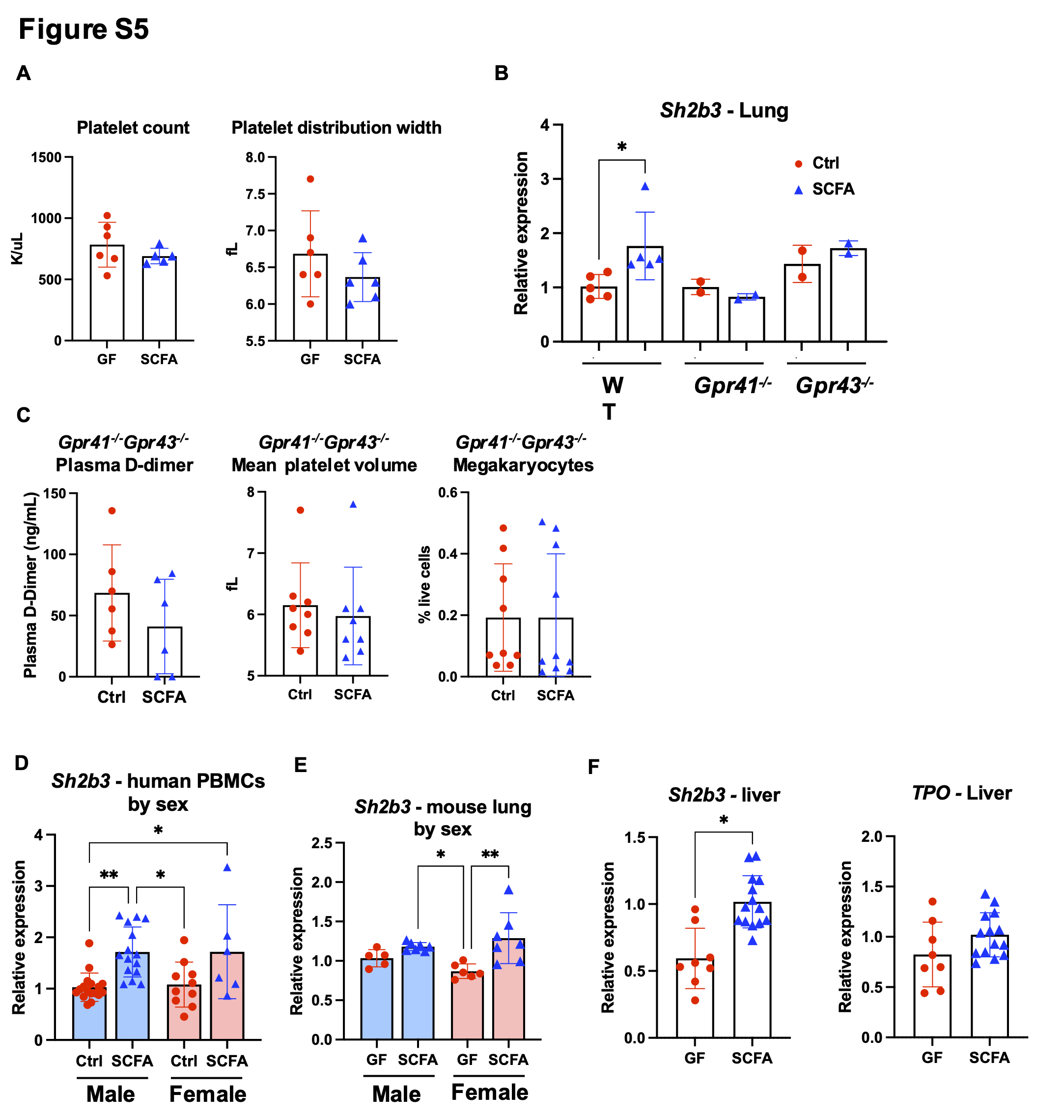

Supplement: Supplemental Material [file KGMI_A_2105609_SM2717.zip › Figure S5.tiff]
